# Is the tungsten(IV) complex (NEt_4_)_2_[WO(mnt)_2_] a functional analogue of acetylene hydratase?

**DOI:** 10.3762/bjoc.13.230

**Published:** 2017-11-02

**Authors:** Matthias Schreyer, Lukas Hintermann

**Affiliations:** 1 Department Chemie, Technische Universität München, Lichtenbergstr. 4, 85748 Garching bei München, Germany; 2 TUM Catalysis Research Center, Ernst-Otto-Fischer-Str. 1, 85748 Garching bei München, Germany

**Keywords:** acetylene hydratase, alkynes, catalytic hydration, enzyme models, tungsten complexes

## Abstract

The tungsten(IV) complex (Et_4_N)_2_[W(O)(mnt)_2_] (**1**; mnt = maleonitriledithiolate) was proposed (Sarkar et al., *J. Am. Chem. Soc.* **1997**, *119*, 4315) to be a functional analogue of the active center of the enzyme acetylene hydratase from *Pelobacter acetylenicus*, which hydrates acetylene (ethyne; **2**) to acetaldehyde (ethanal; **3**). In the absence of a satisfactory mechanistic proposal for the hydration reaction, we considered the possibility of a metal–vinylidene type activation mode, as it is well established for ruthenium-based alkyne hydration catalysts with anti-Markovnikov regioselectivity. To validate the hypothesis, the regioselectivity of tungsten-catalyzed alkyne hydration of a terminal, higher alkyne had to be determined. However, complex **1** was not a competent catalyst for the hydration of 1-octyne under the conditions tested. Furthermore, we could not observe the earlier reported hydration activity of complex **1** towards acetylene. A critical assessment of, and a possible explanation for the earlier reported results are offered. The title question is answered with "no".

## Introduction

**Scheme 1:**
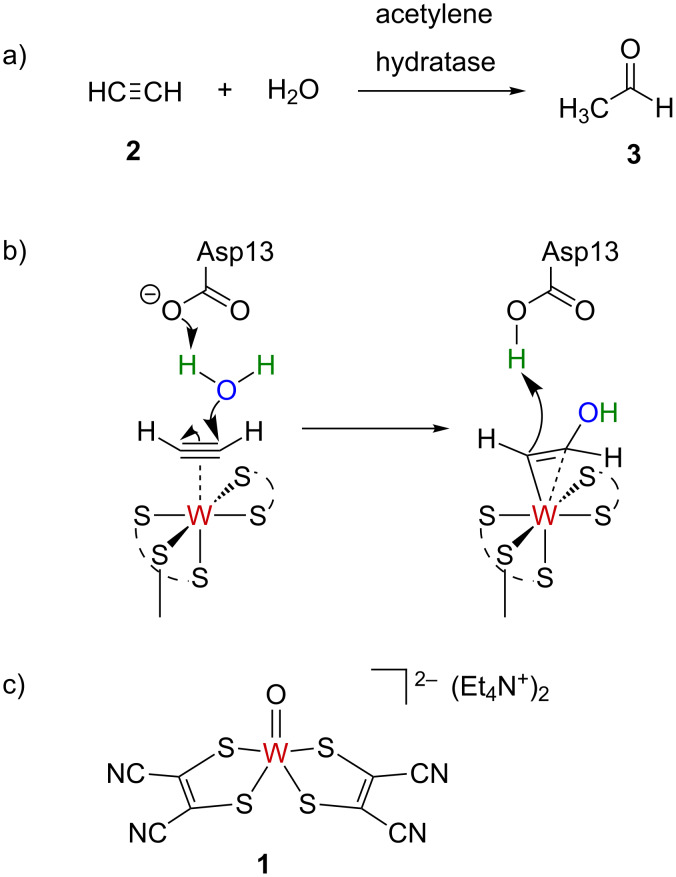
a) Acetylene hydratase catalyzes the hydration of acetylene to ethanal. b) Currently favored key-steps for the reaction mechanism of acetylene hydratase [10–12]. c) Tungsten complex (NEt_4_)_2_[WO(mnt)_2_] (**1**), which was reported as acetylene hydration catalyst [13].

In 1985, the enzyme acetylene hydratase (classification: hydro-lyases, EC 4.2.1) was isolated from the bacterium *Pelobacter acetylenicus* [1], which feeds anaerobically on acetylene as sole carbon source [2]. The enzyme is a tungsten iron–sulfur protein requiring a strongly reducing environment for converting acetylene (ethyne; **2**) to acetaldehyde (ethanal; **3**) by redox-neutral addition of water (Scheme 1a) [2–6]. Even based on X-ray structural data of the enzyme [4], the catalytic reaction mechanism was not immediately obvious [4,7]. Several mechanisms have so far been considered and investigated in silico [8–12]. The most recent works favor nucleophilic addition of water to tungsten-coordinated **2** with assistance of a catalytic carboxylate as key-step (Scheme 1b), followed by protonation of the intermediary 2-hydroxyethenyltungstate to release vinyl alcohol [10–12]. In 1997, Sarkar et al. reported that the oxidation-sensitive but water-stable tungsten(IV) complex (Et_4_N)_2_[W(O)(mnt)_2_] (**1**) (mnt = maleonitriledithiolate) is a catalyst for hydration of acetylene to acetaldehyde, with 9 turnovers over 4 h at ambient temperature (Scheme 1c) [13].

**Scheme 2:**
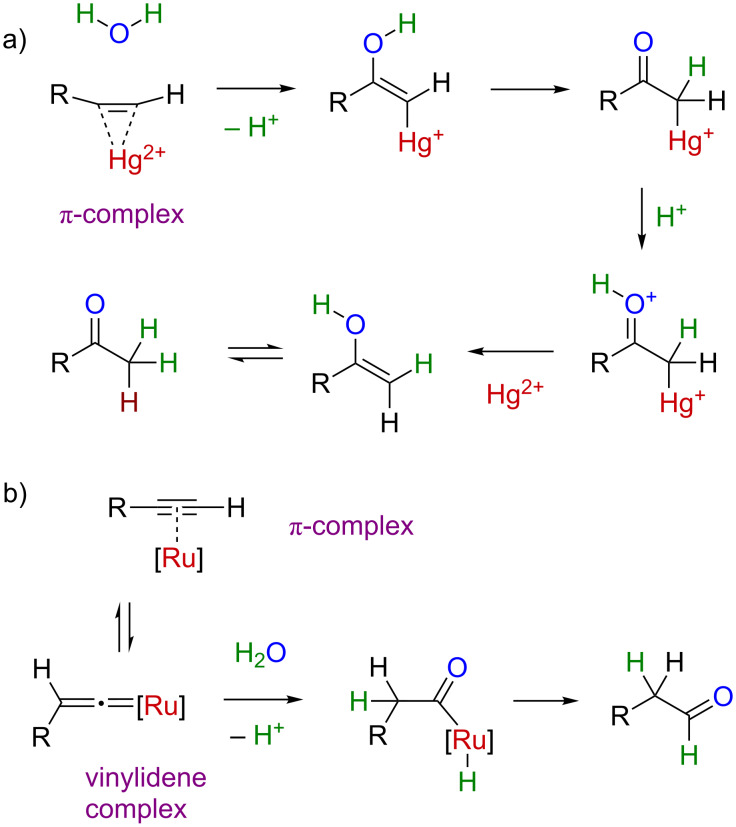
a) π-Activation pathway in Markovnikov selective alkyne hydration, e.g., with mercury catalysts. b) Ruthenium-catalyzed anti-Markovnikov hydration via key vinylidene intermediate.

Tungsten(IV) complex **1** with its two dithiolate ligands that resemble the natural pyranopterindithiolate cofactor ligand [14–16] was suggested to be a functional mimic of the enzyme and a tool to experimentally investigate the mechanism of tungsten-catalyzed acetylene hydration, and by extension the enzymatic reaction mechanism [13]. A theoretical study has considered water addition to coordinated ethyne in [W(η^1^-OH)(mnt)_2_(η^2^-C_2_H_2_)]^–^ with general base activation through the hydroxo ligand and found a reaction pathway with an energy barrier of 20 kcal/mol [17]. Prior to **1**, no molecular tungsten compound had been reported to catalyze alkyne hydration [18], but W(CO)_6_ catalyzes the related cycloisomerization of alkynols, in which the alcohol adds to the alkyne [19]. The reaction of [W(CO)_5_(THF)] with *ortho*-ethynylacetophenone and excess water gives 1,2-diacetylbenzene via neighboring group attack to complexed alkyne, and hydrolysis [20]. The latter pathway represents the π-activation pathway of alkyne hydration (Scheme 2a), whereas alkynol cycloisomerization proceeds via rearrangement to a tungsten vinylidene complex and addition of the alcohol hydroxy group to the vinylidene α-carbon [18].

The vinylidene mechanism is related to that of ruthenium-catalyzed anti-Markovnikov hydration of terminal alkynes to aldehydes (Scheme 2b) [21–23]. Thus, we wondered if tungsten complex **1**, and by analogy acetylene hydratase, is an alkyne hydration catalysts that follows a vinylidene–metal mechanism. This idea has also been considered by others [7–10] and was investigated in silico by Hillier and co-workers [9]. Experimentally, the vinylidene mechanism is revealed in the hydration of a terminal alkyne by producing an aldehyde (anti-Markovnikov type addition) as opposed to a methyl ketone (Markovnikov type addition; typical for π-activation mechanisms) [18]. Hydration reactions of **1** involving higher alkynes have not been reported [13] and substrate scope tests for acetylene hydratase have so far failed with higher alkynes [6]. We wished to test the potential activity and regioselectivity of complex **1** for hydration of higher terminal alkynes, as an extension to our studies of ruthenium-catalyzed anti-Markovnikov hydration [18,24–28].

## Results and Discussion

**Scheme 3:**
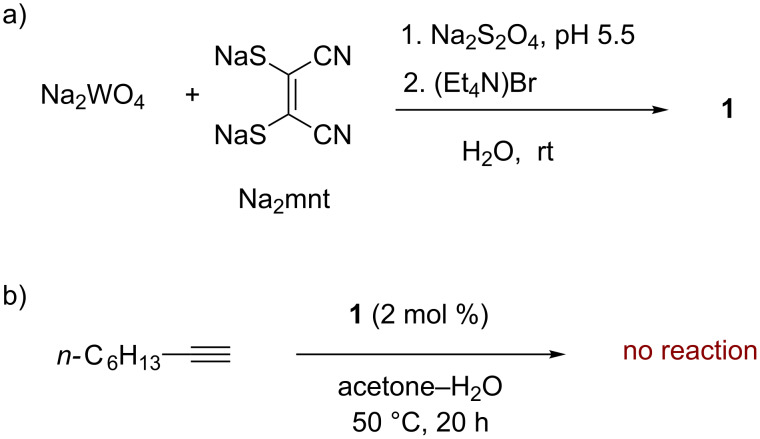
a) Synthesis of complex (NEt_4_)_2_[WO(mnt)_2_] (**1**) [29]. b) Attempted catalytic hydration reaction with a terminal alkyne.

Tungsten complex (NEt_4_)_2_[WO(mnt)_2_] (**1**) was prepared according to the literature procedure from Na_2_WO_4_, Na_2_mnt and buffered aqueous dithionite, followed by precipitation with Et_4_NBr (Scheme 3a) [29]. The compound was characterized by ^1^H and ^13^C NMR spectroscopy, IR spectroscopy, and by its dark purple color. A diagnostic analytical property in solution is δ_C_ of C-2/3 in the mnt ligand (δ_C_ = 140.4 ppm for **1**) that depends on changes in the oxidation state, particularly oxidation to [WO_2_(mnt)_2_]^2−^ (δ_C_ = 123.3 ppm).

In a preliminary experiment, 1-octyne and complex **1** (2 mol %) were heated at 50 °C in aqueous acetone. In situ analysis of the reaction mixture after 20 hours by GC–MS failed to reveal any new product next to unchanged 1-octyne.

We have recently developed standardized screening procedures for detecting alkyne hydration activity and regioselectivity of potential catalysts. The test system is based on heating substrate 10-undecyn-1-ol (**4**) together with a potential catalyst in degassed water–solvent mixtures to 160 °C for 15 min in a microwave reactor (Table 1). Analysis of the reaction mixture by ^1^H NMR against internal standard reveals conversion and product selectivity (**5** vs **6**), or points to important side-reactions through spectroscopic identification and quantification of side-products. Extensive catalyst screening studies that also included established alkyne hydration catalysts have shown that every single of the known catalyst shows significant activity under the conditions of this test [30]. The first two entries in Table 1 exemplify the performance of typical alkyne hydration catalysts with Markovnikov ([AuPPh_3_]^+^, entry 1; product **5**) or anti-Markovnikov selectivity (entry 2; product **6**) in the screening setup.

**Table 1:** Hydration experiments with 10-undecyn-1-ol (**4**).^a^

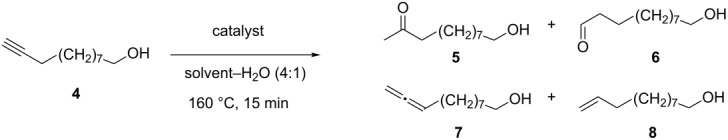								

Entry	Catalyst (mol %)	Solvent^b^	**4** (%)	**5** (%)	**6** (%)	**7** (%)	**8**^c^ (%)	Recovery (%)

1	AuClPPh_3_ (2)	MeOH	57.7	40.7	0.4	0.8	0	100.0^d^
2	CpRuCl(PPh_3_)_2_–ISIPHOS (2)	acetone	0.0	0.1	91.2	0	1.5	92.8
3^e^	**1** (20)	acetone	96.8	n.d.^f^	0.7	n.d.^f^	n.d.^f^	97.5
4^e^	–	acetone	88.4	0.8	1.1	0.8	0	91.1
5	–	acetone	97.1	0.0	0.3	0.8	1.2	99.4
6	**1** (20)	acetone	97.8	0.0	0.1	0.7	1.4	100.0
7	**1** (20)	MeCN^g^	97.1	0.0	0.2	0.5	1.5	99.3
8^h^	**1** (20)	acetone	97.8	0.0	0.2	0.8	1.7	100.5

^a^Reaction conditions: microwave heating, 160 °C, 15 min. Composition of crude product is given in mol % relative to initial **4**, as determined by qNMR against internal standard. Recovery is the sum of analytically detected **4** and products derived from it. ^b^Solvent and water were applied in a 4:1 volume ratio, unless otherwise mentioned. ^c^May include other alkanols, like undecan-1,11-diol or 1-decanol. ^d^Includes 0.4 mol % of the dimethylacetal of **5**. ^e^Non-distilled starting material **4** was used. ^f^A low signal-to-noise ratio prevented reliable detection of **5**, **7** and **8**, which in any case must have been low. ^g^Solvent–H_2_O volume ratio 2:1. ^h^Reaction performed in a Schlenk tube at 60 °C for 24 h. The higher than 100% recovery reflects experimental error.

**Scheme 4:**
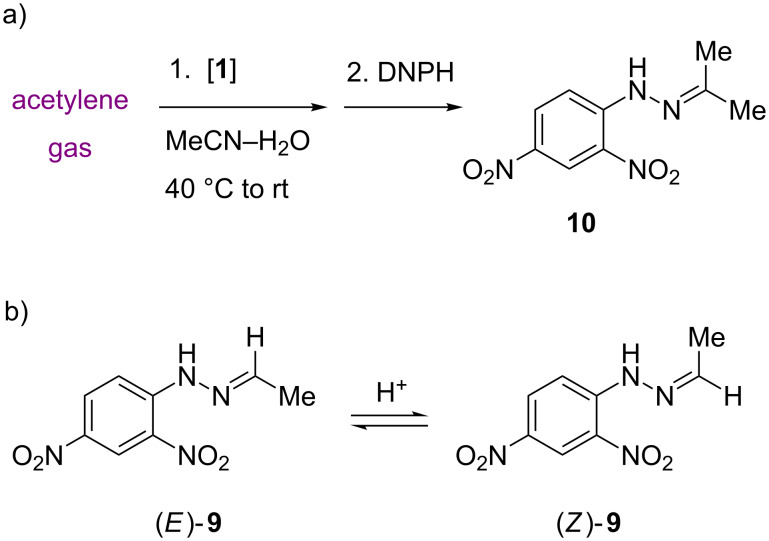
a) Unexpected isolation of acetone 2,4-dinitrophenylhydrazone (**10**) from an attempted catalytic hydration of ethyne (acetylene gas) in the presence of **1**. b) Acetaldehyde 2,4-dinitrophenylhydrazone (**9**) exists as two geometrical isomers. DNPH = 2,4-dinitrophenylhydrazine.

More specifically, gold complex AuCl(PPh_3_) is not usually considered an alkyne hydration catalyst itself, but turns into a catalytically active gold(I) cation after activation with silver salt or Lewis acid [31–34]. Under the forcing microwave reaction conditions in aqueous methanol, ionization is brought about without an added reagent, and catalytic activity towards Markovnikov product **5** revealed, even if the conversion is low (Table 1, entry 1). A cyclopentadienylruthenium(II) catalyst with the ambifunctional steering ligand ISIPHOS (2-(diphenylphosphino)-6-(2,4,6-triisopropylphenyl)pyridine) [24–26,28] expectedly brings about anti-Markovnikov hydration, with aldehyde **6** as major product. Table 1, entry 3 represents a first test of tungsten complex **1** in the microwave hydration protocol, at a fairly high catalyst loading of 20 mol %. Besides unchanged **4**, the reaction mixture contained 0.7% of aldehyde **6**. Could this mean that **1** is indeed an anti-Markovnikov hydration catalyst? The low turnover number of 0.035 implies that this is unlikely. A blank experiment revealed that even higher amounts of **6** are generated from **4** in the absence of catalyst (Table 1, entry 4). Aldehyde **6** is an impurity in the starting alkyne **4**, but may also be formed under the reaction conditions if the latter contains autoxidation impurities. Kugelrohr vacuum distillation of **4** reduced the aldehyde level to below 0.3% (Table 1, entries 5–8). Even if technically unsatisfactory, entries 3 and 4 are included in Table 1 to illustrate a potential pitfall in the study of catalytic oxyfunctionalization of unsaturated hydrocarbons, where autoxidation products may feign false positive results [35]. The effect is most problematic at low mol % loadings, and it is thus necessary to substantiate a presumed catalytic activity by increasing conversion to higher levels through increasing the catalyst loading [36]. Tests of complex **1** with purified **4** in acetone–water or acetonitrile–water failed to show catalytic activity (Table 1, entries 6 and 7). The recovery of **4** was excellent (97–98%) and the side products allenol **7** and alkenol **8** are impurities already present in distilled starting material. Thus, complex **1** does not show hydration activity against higher terminal alkynes. To further validate those negative results, we wished to demonstrate a positive activity of complex **1**, namely the reported hydration of ethyne to acetaldehdye [13]. A commercial acetylene pressure bottle (purity grade 2.6, i.e., 99.6%, containing ≤5 ppm sulfur or phosphorus compounds) was available for the experiment. Gas was first bubbled through a solution of **1** in acetonitrile–water (2:1) at 40 °C, then the vessel was closed and the reaction mixture incubated at room temperature. Derivatization of the reaction solution with 2,4-dinitrophenylhydrazine (DNPH) precipitated a yellow substance. The original report had identified the precipitate as acetaldehyde 2,4-dinitrophenylhydrazone (**9**) by recording a melting point (147 °C) and determining an HPLC peak retention time against reference material. Since neither analytical method provides structural information, we analyzed the product by ^1^H NMR spectroscopy instead and found to our initial surprise that the precipitated material was the dinitrophenylhydrazone **10** derived from acetone, with no acetaldehyde hydrazone **9** present (Scheme 4a)!

**Figure 1:**
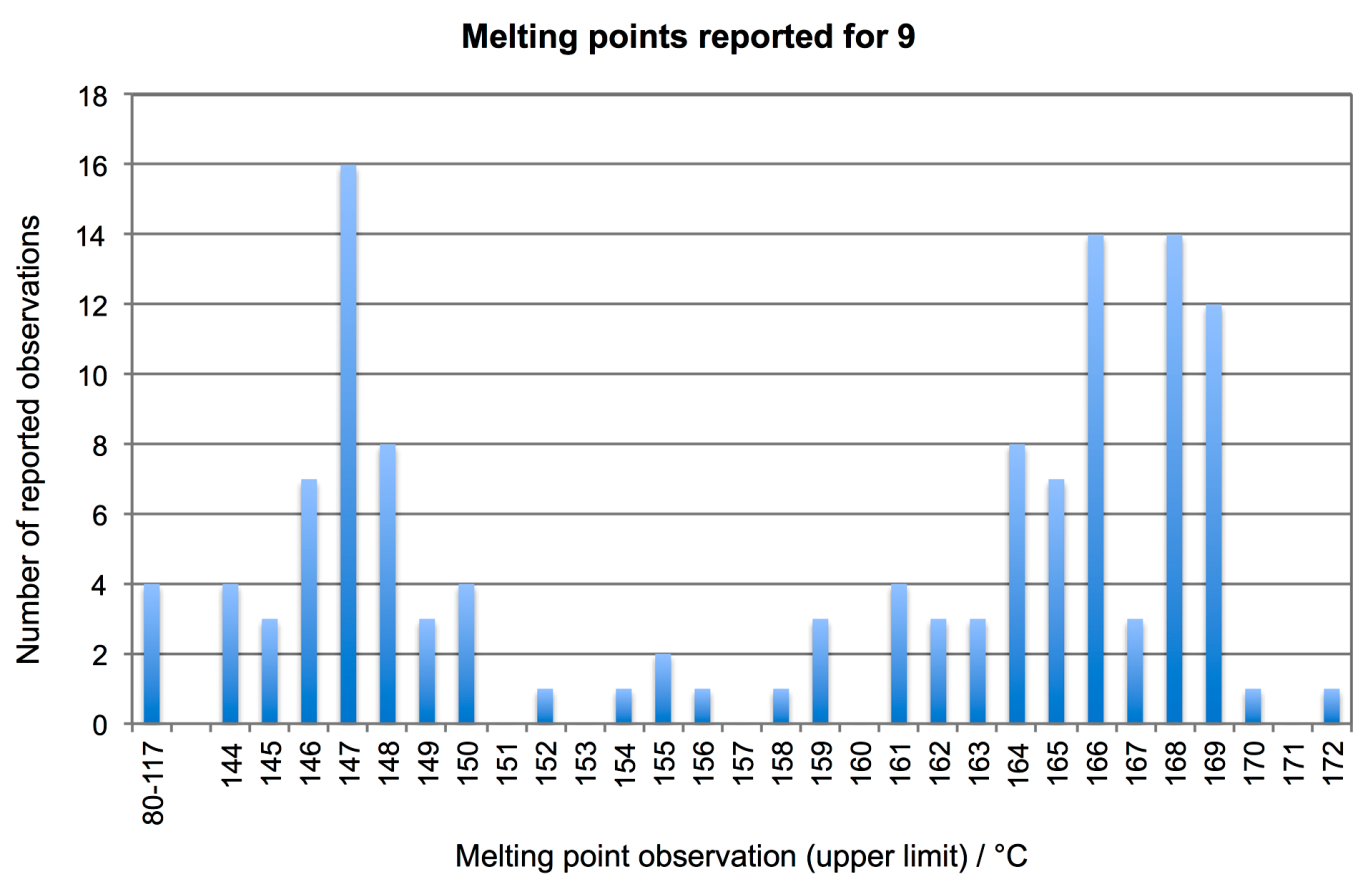
Frequency of reported melting points for acetaldehyde 2,4-dinitrophenylhydrazone (**9**) from the Reaxys database (June 2017). Where a melting-range had been reported, the upper limit was chosen for the analysis.

Retrospectively, this result could have been expected, since acetone is present as stabilizer in commercial acetylene pressure bottles [37]. The original report on **1**-mediated acetylene hydration did not consider (and thus did not exclude) the generation of **10**, and the source and purification method for substrate **2** were not indicated [13]. Derivatization to 2,4-dinitrophenylhydrazones is a well-established identification method for carbonyl compounds that recommends itself for small amounts of volatile products [38–39]. However, aldehyde dinitrophenylhydrazones often exist as mixtures of *E* and *Z*-isomers (Scheme 4b), which interconvert in the presence of co-precipitated acid, and stable melting points cannot be achieved, unless special purification protocols are followed [40–41]. The inadequacy of identifying **9** by standard melting point measurements is emphasized by the histogram in Figure 1 which was created from melting point data in the Reaxys database and shows scattering over 30 °C.

Consequently, measuring a single or even a mixed melting point with a reference sample is not a reliable identification criterion for **9**. An analysis of the compound by HPLC had also been performed, where a peak retention time (3.072 min) was given for the product, together with some specifics of the analytical setup, but without identifying the analytical column. In the absence of proof that mixed injections of **9** and **10** would give rise to separate peaks, the HPLC test cannot be considered to identify **9** or differentiate it from **10**. In short, we find that the analytical evidence presented in ref. [13] to identify **9** was insufficient, and thus the catalytic hydration of **2** to **3** by complex **1** is not proven. In particular, the possibility that acetone was mistaken for **3** cannot be excluded.

**Figure 2:**
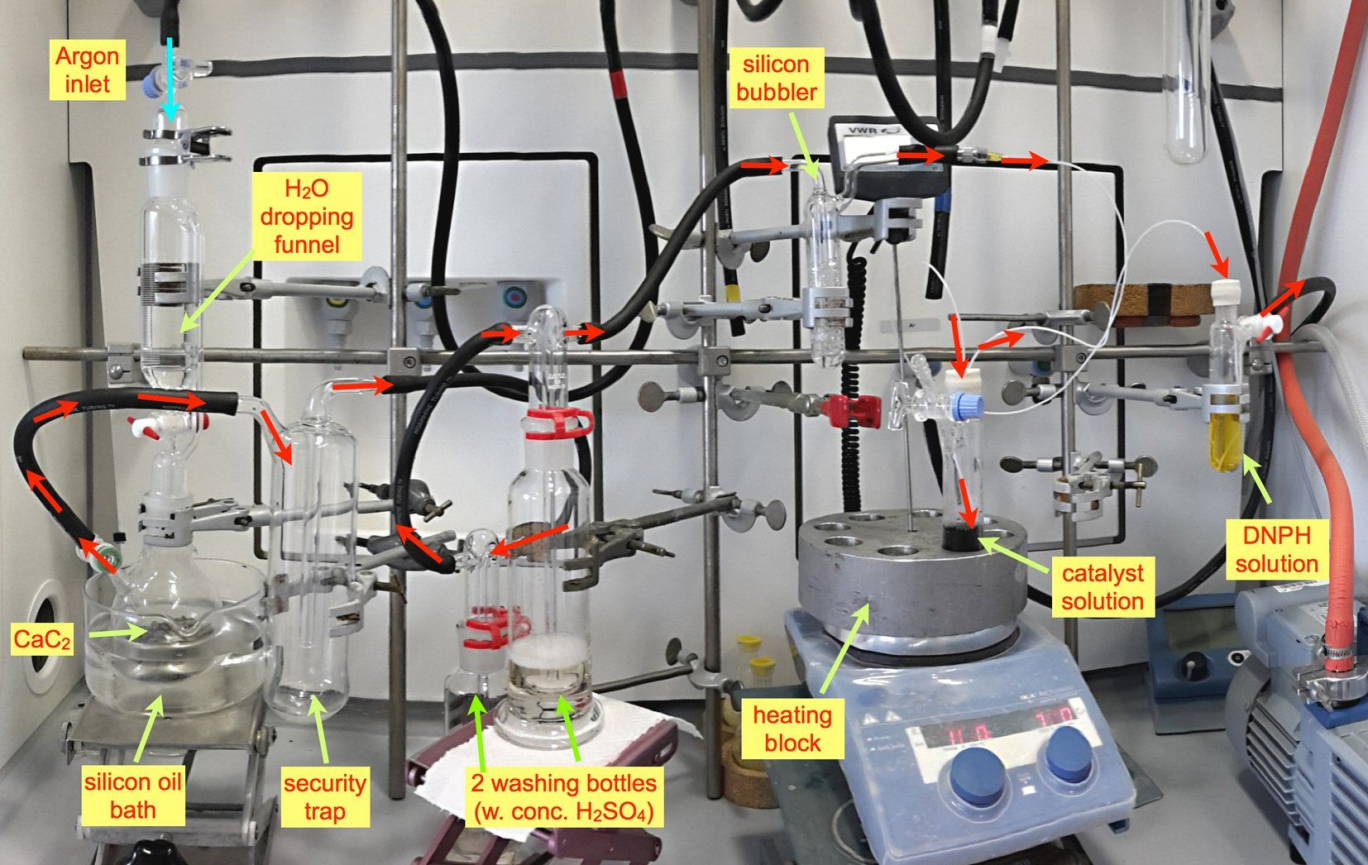
Experimental setup for the study of catalytic acetylene hydration. Red arrows indicate the direction of the gas flow.

Continued interest in functional models of acetylene hydratase [14–16,42] and a theoretical study on the mechanisms of acetylene hydration by complex **1** [17] have motivated us to scrutinize the claimed biomimetic catalytic activity by performing the hydration experiment with acetone-free **2**. A stream of gas was generated by dropping water on calcium carbide (CaC_2_) and passed through two washing bottles with concentrated sulfuric acid to remove polar impurities (Figure 2).

**Figure 3:**
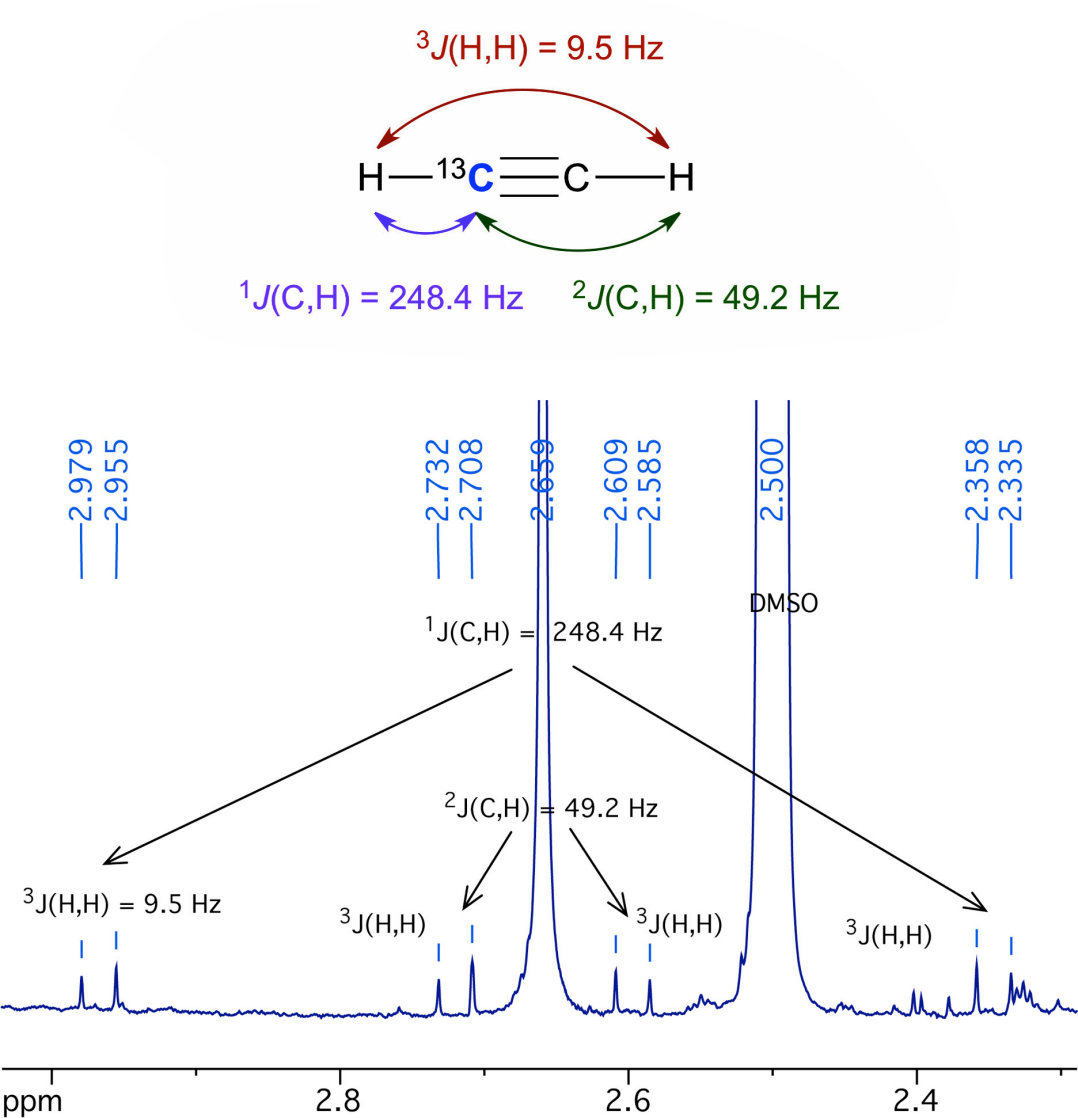
Identification of ethyne (**2**) in the reaction solution by coupling pattern analysis of ^13^C-satellite signals.

Oxygen was carefully excluded from the reaction system to prevent oxidation of **1** to inactive the tungsten(VI) species [13]. The experiment was carried out either by incubating catalyst **1** in acetylene-saturated aqueous acetonitrile (static conditions), or by prolonged bubbling of a stream of **2** through the catalyst solution (dynamic conditions) [13]. In the latter case, the exhaust gas was bubbled through an acidic solution of DNPH to absorb volatile carbonyl compounds. Emphasis was placed both on unequivocal and direct analysis of acetaldehyde (**3**) in the reaction solution, and on identification of all major species in the reaction solution or the DNPH solution. For this purpose, ^1^H and ^13^C NMR analyses of the reaction (catalyst) solutions were performed with addition of DMSO-*d*_6_ for locking. The components detected in the reaction solution were catalyst **1** by δ_H_ 1.14 and 3.15 for the tetraethylammonium cation, and δ_C_ 119.5, 141.0 for the mnt ligand, which is characteristic for the tungsten complex and proves that **1** was intact throughout the reaction. Signals for **2** were detected at δ_H_ 2.66 and δ_C_ 75.1; the identity as acetylene was proven beyond doubt by analyzing the ^13^C,^1^H coupling pattern from the ^13^C satellites in the ^1^H NMR spectrum (Figure 3) [43–44].

The concentration of **2** in the final reaction solution was determined to 0.1 mol/L. No new compounds could be detected in appreciable amounts. Acetaldehyde in particular was absent, with an estimated limit of detection corresponding to 0.05 turnovers. The result was the same in the dynamic (bubbling) or static (incubation) experiment. No carbonyl hydrazones were detected in the DNPH absorption solutions or the reaction solutions after treating with acidic DNPH by recording ^1^H NMR spectra of the precipitates or filtrates after evaporation to dryness. Only unchanged DNPH was detected.

Since the catalysis had given a negative result, we felt it important to ascertain that we would have detected acetaldehyde, had it formed under reaction conditions. As a test of the analytical procedures, a hydration experiment with acetylene gas was performed in the same setup (Figure 2), but with CpRuCl(PPh_3_)_2_–ISIPHOS as established anti-Markovnikov alkyne hydration catalyst (cf. Table 1) [24]. The catalyst solution was prepared in aqueous triethyleneglycol dimethyl ether (triglyme), because acetonitrile is a competitive inhibitor of the ruthenium catalyst [24], and acetone would have disturbed the DNPH-test for acetaldehyde. Bubbling acetylene through the solution for 4 h at 35 °C produced plenty of precipitate in the DNPH-exhaust solution, which was shown to be acetaldehyde dinitrophenylhydrazone **9** by ^1^H and ^13^C NMR spectroscopy. The NMR spectrum of the catalyst solution contained distinct signals for acetaldehyde (δ_H_ 2.11, 9.64; ^3^*J*(H,H) = 9.6 Hz), but their rather low intensity indicates that product vapors are efficiently transferred further into the DNPH exhaust solution by the acetylene stream.

## Conclusion

Acetylene hydratase is a fascinating enzyme that catalyzes the hydration of ethyne to ethanal (acetaldehyde) by what appears to be a nucleophilic mechanism with assistance by carboxylate base. As such, the mechanism is related to Reppe's alkali-mediated addition of alcohols to acetylene that gives vinyl ethers [45]. Alkali-mediated hydration of alkynes is not known for regular alkynes, since the carbonyl reaction products are unstable to the strongly alkaline reaction medium [18]. Base-mediated hydration is possible for π-acceptor substituted alkynes, but nucleophilic addition of secondary amines followed by acidic hydrolysis to the carbonyl compound is usually preferred [18]. The discovery of a tungsten-based enzyme with ethyne hydration activity was quite surprising and the enzymatic reaction mechanism was not immediately evident, for simple tungsten complexes had not been known to bring about alkyne hydration. The situation changed 1997 by a report of Sarkar et al. who described the activity of complex **1** for acetylene hydration [13]. This observation was at the same time remarkable in terms of a new reactivity and appeared to immediately "explain" the enzymatic reaction as primarily metal-based. Through our writing of a review article on alkyne hydration chemistry [18] as well as our work in ruthenium-catalyzed anti-Markovnikov hydration of terminal alkynes [24–28] we became aware of this chemistry and considered the fascinating possibility of an enzymatic vinylidene-type alkyne hydration mechanism. If applicable, this would have opened new possibilities for catalytic anti-Markovnikov hydration of alkynes too, which is currently limited to ruthenium(II) complexes [18]. Unfortunately, neither was complex **1** active in the hydration of higher, terminal alkynes, nor could we reproduce the originally reported hydration of acetylene. The experiments presented here exclude generation of acetaldehyde from ethyne under the reaction conditions, even in trace amounts. They also highlight potential difficulties in the analytical detection of acetaldehyde and emphasize the problem of potential contamination of acetylene by acetone, which is used as stabilizer in commercial acetylene pressure gas bottles. Conclusively, the title question can be answered with "no".

## Supporting Information

File 1 Experimental procedures and NMR spectra.
