# Supplementary material for: Is the tungsten(IV) complex (NEt4)2[WO(mnt)2] a functional analogue of acetylene hydratase?
Source: Beilstein J Org Chem. 2017 Nov 2;13:2332–9. doi: 10.3762/bjoc.13.230 (PMC5687055; doi:10.3762/bjoc.13.230)

Supporting Information

for

Is the tungsten(IV) complex $(NEt_4)_2[WO(mnt)_2]$ a functional analogue of acetylene hydratase?

Matthias Schreyer[1,2] and Lukas Hintermann*[1,2]

Address: [1]Department Chemie, Technische Universität München, Lichtenbergstr. 4, 85748 Garching bei München, Germany and [2]TUM Catalysis Research Center, Ernst-Otto-Fischer-Str. 1, 85748 Garching bei München, Germany

Email: Lukas Hintermann - lukas.hintermann@tum.de

* Corresponding author

Experimental procedures and NMR spectra

Table of contents

1 General **S3**

2 Experiments **S4**

2.1 Starting materials S4

2.1.1 Tungsten complex $(NEt_4)_2[WO(mnt)_2]$ S4

2.1.2 10-Undecyn-1-ol (**4**) S4

2.2 Catalytic alkyne hydration under microwave conditions S5

2.2.1 General experimental procedure for catalytic microwave hydrations S5

2.2.2 Product analysis by quantitative ^{1}H NMR spectroscopy S5

2.3 Attempted catalytic hydration of 1-octyne with tungsten complex **1** S5

2.4 Ethyne (acetylene) hydration experiments with tungsten complex **1** S7

2.4.1 Experiments using acetylene gas generated from calcium carbide S7

2.4.2 Experiments using acetylene gas from a commercial pressure gas bottle S9

2.4.3 Acetylene hydration with a $CpRuCl(PPh_3)_2$–ISIPHOS catalyst S10

3 NMR spectra **S12**

3.1.1 $(NEt_4)_2[WO(mnt)_2]$ (**1**) S12

3.1.2 Acetylene (**2**) hydration experiments S13

3.1.3 Acetaldehyde (**3**) reference spectrum in D_2O S15

3.1.4 10-Undecyn-1-ol (**4**) S16

3.1.5 Microwave catalytic hydration screening with $AuCl(PPh_3)$ - crude reaction mixture S17

3.1.6 Microwave catalytic hydration screening with $CpRuCl(PPh_3)_2$–ISIPHOS - crude S17

3.1.7 Acetaldehyde 2,4-dinitrophenylhydrazone (**9**) S18

3.1.8 Acetone 2,4-dinitrophenylhydrazone (**10**) S19

1 General

Abbreviations

DNB	1,3-dinitrobenzene
DMF	*N*,*N*-dimethylformamide
DNP	2,4-dinitrophenyl
DNPH	2,4-dinitrophenylhydrazine (*or:* 2,4-dinitrophenylhydrazone)
Et_2O	diethyl ether
mnt	(*Z*)-1,2-dicyanoethene-1,2-bis(thiolate) (*or*: maleonitrile-dithiolate)
THF	tetrahydrofuran
TLC	thin-layer chromatography
TMS	tetramethylsilane
triglyme	triethylene glycol dimethyl ether

2 Experiments

2.1 Starting Materials

2.1.1 Tungsten complex $(NEt_4)_2[WO(mnt)_2]$

Complex $(NEt_4)_2[WO(mnt)_2]$ was synthesized from $Na_2(mnt)$[1] according to the literature procedure.[2] The material was recrystallized from MeCN/Et_2O.

^{1}H NMR ((D$_6$)-DMSO, 360 MHz, 300 K): δ 1.15 (tt, ^3J(H,H) = 7.3 Hz, ^3J(^{14}N,^{1}H) = 1.85 Hz, 24 H, C*H*$_3$), 3.19 (q, ^3J(H,H) = 7.3 Hz, 16 H, C*H*$_2$). **^{13}C{^{1}H} NMR**: ((D$_6$)-DMSO, 91 MHz, 300 K): δ 7.04 (CH_3), 51.40 (t, ^1J(^{14}N,^{13}C) = 3.0 Hz, CH_2; Et_4N^+), 119.01 (C≡N, mnt), 140.37 (C, mnt). **IR (ATR)**: ν/cm^{-1} = 2192 (vs, C≡N), 1481 (vs, C=C), 934 (vs, W=O).

2.1.2 10-Undecyn-1-ol (4)

The commercial product (Alfa, 96% purity) was purified by Kugelrohr short-path vacuum distillation. The purity of the material was assessed by quantitative ^{1}H NMR spectroscopy.

Table S-1. Analysis of distilled 4[a]

component	^{1}H NMR signals	[wt-%]
10-undecyn-1-ol (**4**) 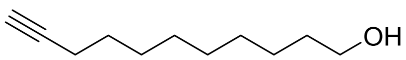	δ 1.94 (t, J = 2.7 Hz, 1 H), 2.18 (td, J = 7.1, 2.7 Hz, 2 H)	98.2
9,10-undecadien-1-ol (**7**) 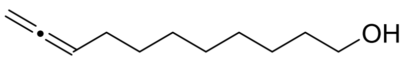	δ 4.64 (dt, J = 6.7, 3.2 Hz, 2 H), 5.09 (quint, J = 6.7 Hz, 1 H)	0.8
11-hydroxyundecanal (**6**) 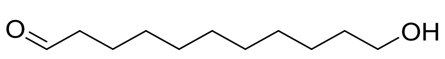	δ 9.76 (t, J = 1.9 Hz, 1 H)	0.1
undec-9-yn-1-ol 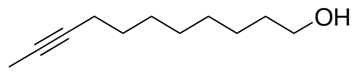	δ 1.78 (t, J = 2.6 Hz, 3 H)	0.3

a) Sample: 20.9 mg; internal standard 1,3-dinitrobenzene: 10.2 mg, 60.68 μmol; measured in $CDCl_3$.

---

[1] a) G. Bähr, G. Schleitzer, *Chem. Ber.* **1955**, *88*, 1771–1777. b) G. Bähr, G. Schleitzer, *Chem. Ber.* **1957**, *90*, 438–443. c) R. H. Holm, A. Davison, *Inorg. Synth.* **1967**, *10*, 8–26.

[2] S. K. Das, D. Biswas, R. Maiti, S. Sarkar, *J. Am. Chem. Soc.* **1996**, *118*, 1387–1397.

2.2 Catalytic alkyne hydration under microwave conditions

2.2.1 General experimental procedure for catalytic microwave hydrations

A 10 mL microwave vial with magnetic stirring bar was thrice evacuated and argon backfilled. Under argon, degassed water (0.5 mL), degassed acetone (2.0 mL), catalyst and 10-undecyn-1-ol (20.0 µL, 104 µmol) were added and the vial was capped. The reaction mixture was microwave-heated to 160 °C and stirred for 15 min at target temperature. After cooling, the reaction mixture was transferred into a vial containing a solution of a known amount of 1,3-dinitrobenzene in Et_2O (0.5 mL). The reaction vessel was repeatedly rinsed with Et_2O (3 × 2 mL) and the resulting solution thoroughly homogenized. An aliquot (0.5 mL) was diluted with Et_2O (2 mL), washed with water (3 × 2 mL) and brine (2 mL) and dried over anhydrous $MgSO_4$. After evaporation of the solvent (water jet pump), $CDCl_3$ (0.6 mL) was added to the residue. The homogenized solution was transferred into an NMR tube and analyzed by NMR, using a pulse repetition delay of 20 seconds.

2.2.2 Product analysis by quantitative ^{1}H NMR spectroscopy

The ^{1}H NMR spectrum was Fourier-transformed, phase-corrected and baseline-corrected. The chemical shift was referenced to TMS (δ 0.00).[3] The peak integral of the signal for internal standard 1,3-dinitrobenzene (DNB) (δ 9.08; t, ^4J(H,H) = 2.2 Hz, 1 H; integration excluding ^{13}C-satellites) was set to the molar ratio $n_{(DNB)}/n_{(alkyne)}$.

2.3 Attempted catalytic hydration of 1-octyne with tungsten complex 1

Acetone (4 mL) and water (1 mL) were placed in a Schlenk vessel (10 mL) and degassed by argon sparging at 0 °C; tungsten complex (**1**; 18 mg, 24 µmol) and 1-octyne (160 mg, 1.45 mmol) were added and the resulting purple solution placed into an oil-bath at 50 °C and stirred for 20 h. A sample was removed for GC–MS analysis and only revealed the presence of 1-octyne (Figure S1).

[3] G. R. Fulmer, A. J. M. Miller, N. H. Sherden, H. E. Gottlieb, A. Nudelman, B. M. Stoltz, J. E. Bercaw, K. I. Goldberg, *Organometallics* **2010**, *29*, 2176–2179.

Figure S1: GC–MS trace of 1-octyne hydration experiment with complex 1

```
File       : C:\HPCHEM\1\DATA\LH_XXX.D
Operator   : lh07
Acquired   :  9 Jun 07   1:36 pm using AcqMethod NORMOL
Instrument :    HP 5973 M
Sample Name: hydration tungsten
Misc Info  :
Vial Number: 1
```

V1055

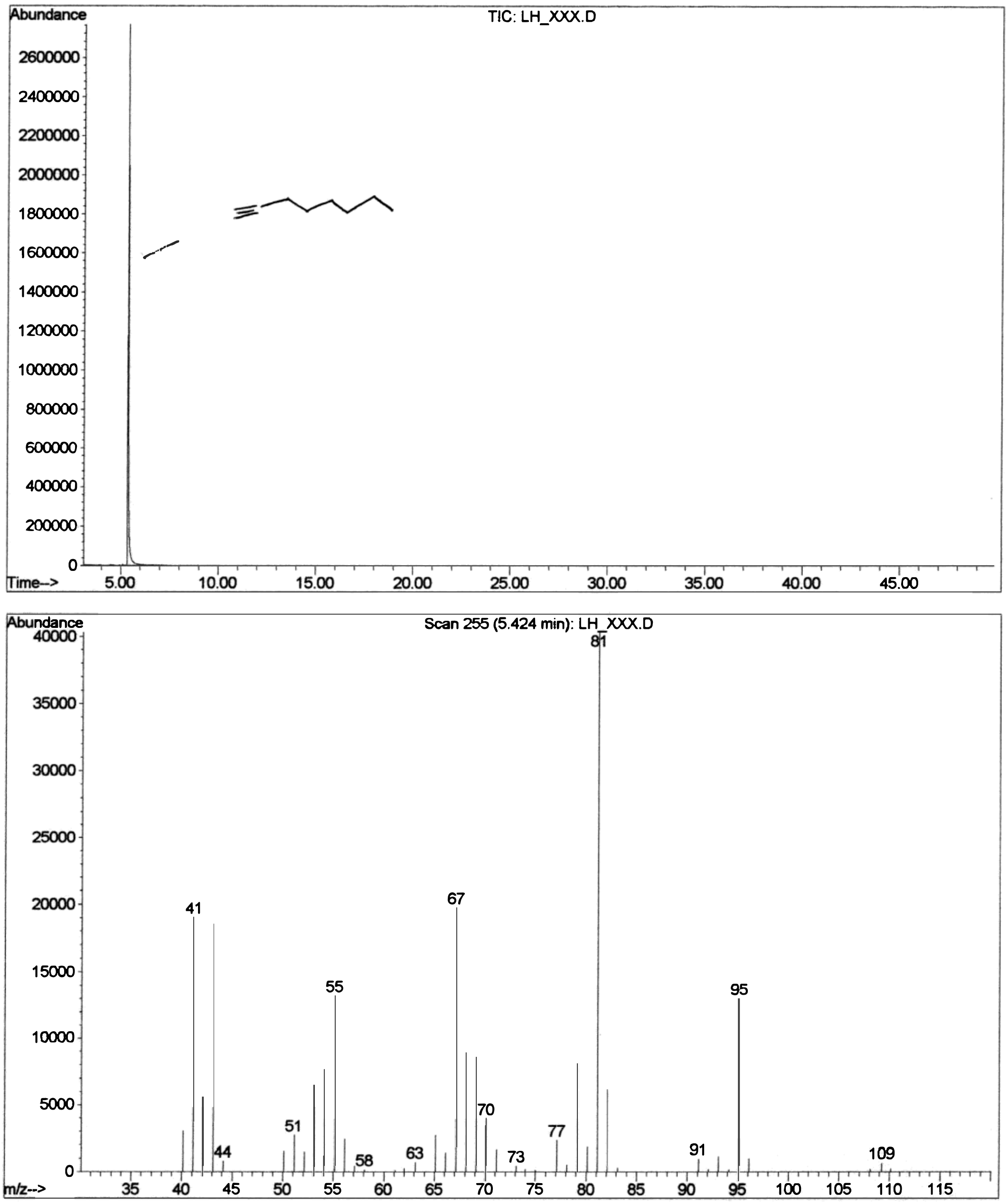

2.4 Ethyne (acetylene) hydration experiments with tungsten complex 1

2.4.1 Experiments using acetylene gas generated from calcium carbide

(Compare Figure S2 for the reaction setup): A 250 mL Schlenk flask was charged with CaC_2 (15–20 g, technical quality) and placed into a silicon oil bath at room temperature for temperature moderation. A dropping funnel was mounted and its top connected to the argon line. The gas-outlet was connected via neoprene tubings to an empty cooling trap (serving as security trap in case of backflush), two washing bottles (with sintered glass filter) containing concentrated H_2SO_4 and a silicon oil bubbler. The bubbler outlet was connected to PTFE tubing (ø 0.8 mm i. d.), which was inserted into the Schlenk reaction vessel (25 mL) through a septum and passed into the catalyst solution. The gas outflow was allowed to run through PTFE tubing through a septum into another Schlenk vessel (25 mL) contain acidic 2,4-DNPH solution (65 mg DNPH in 15 mL 2 M HCl aq) and the end of the tubing placed into the solution. The exhaust gas was let off into the hood ventilation system.

Figure S2: Illustration of a reaction setup (ethyne flow conditions)

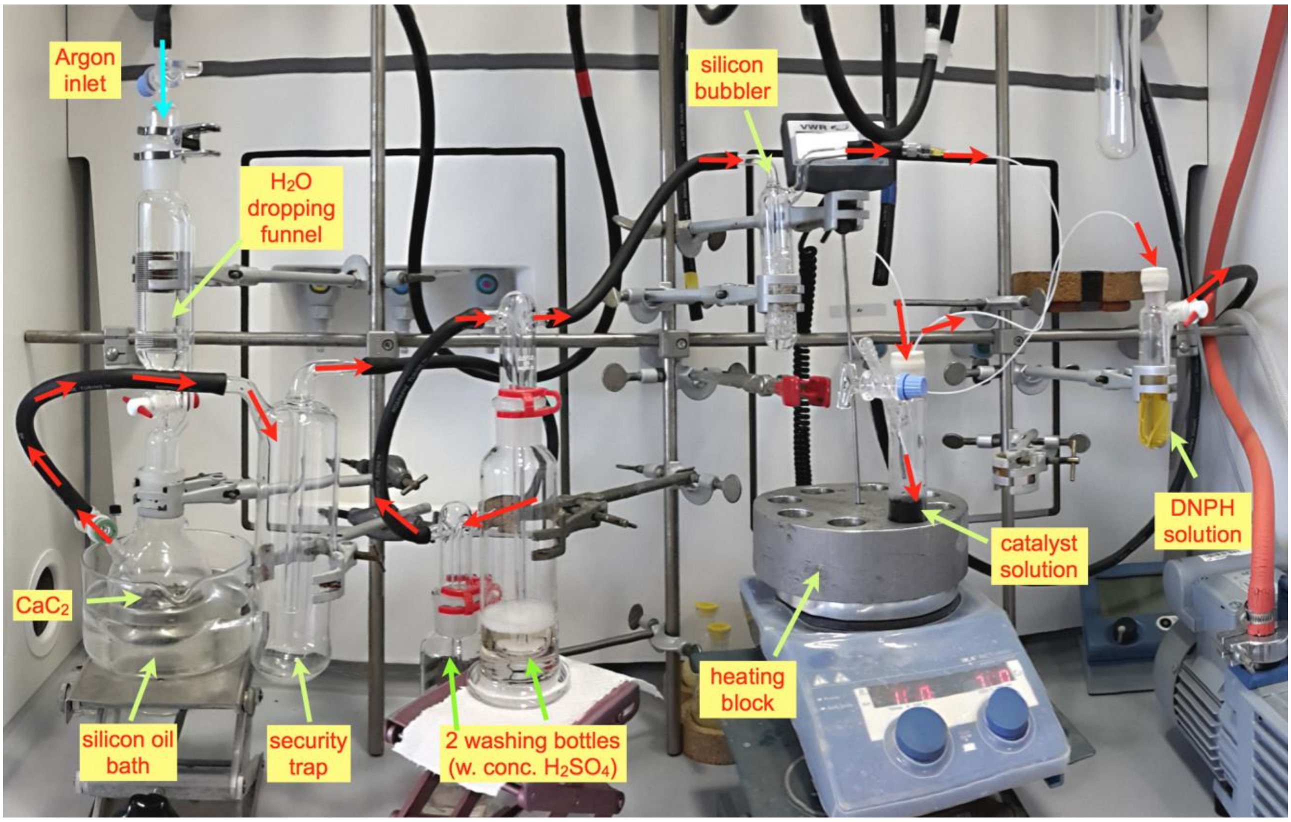

For the "incubation" reaction, the catalyst solution was placed on the bottom of a 250 mL Schlenk vessel which was filled with a pure ethyne atmosphere prior to incubation at 35 °C; since there was no gas-flow during the experiment, no DNPH-absorber vessel for exhaust gas was used.

After charging the apparatus with CaC_2 and degassed H_2O, it was flushed with argon. The reaction flask was then charged with acetonitrile and water, placed in an ice-bath and flushed with argon for ca 20 min. Finally, the catalyst was added to the reactor in an argon counter-stream and acetylene generation was started by ***very careful,*** slow dropping of water on CaC_2. The argon stream was stopped and replaced by a steady flow of acetylene.

a) Experiment with acetylene bubbling

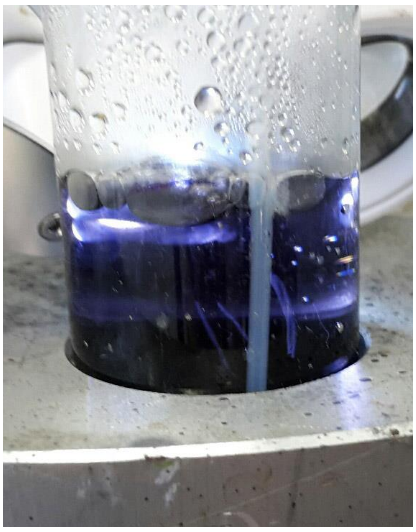

Catalyst solution during bubbling with acetylene.

The reaction flask was charged with complex **1** (100 mg, 135 μmol), acetonitrile (10 mL) and water (10 mL); the solvents were degassed prior to inserting into the reaction system. Once the acetylene flow was steady, the dark purple catalyst solution was removed from the ice-bath and placed in a metal-block at 35 °C. The DNPH solution remained clear for the whole reaction time. After 4 h, the acetylene flow was stopped and the reaction solution cooled to rt. A sample of the still dark purple reaction solution (200 μL) was removed for NMR analysis in (D_6)-DMSO (ca. 400 μL). The exhaust absorption DNPH-solution was neutralized with Na_2CO_3 aq and the resulting suspension was filtered; the solid was washed with water and analyzed by NMR in $CDCl_3$ (low solubility). The filtrate was evaporated by rotatory evaporation in vacuum and the residue was extracted with $CDCl_3$ for NMR analysis. Both the solid and the filtrate only consisted of 2,4-dinitrophenylhydrazine with no presence of DNP-hydrazones.

NMR analysis of the reaction solution: ^{1}H NMR (400 MHz, (D_6)-DMSO): ethyne, δ 2.66 (s, 2 H); MeCN, δ 2.00 (s); H_2O, δ 3.80 (s); NEt_4^+, δ 1.14 (tt, ^3J(H,H) = 7.3 Hz, ^3J(^{14}N,^{1}H) = 1.9 Hz, 24 H, CH_3), 3.14 (d, ^3J(H,H) = 7.3 Hz, 16 H, CH_2). ^{13}C NMR (101 MHz, (D_6)-DMSO): ethyne, δ 75.12 (s); MeCN, δ 1.58, 118.69; NEt_4^+, δ 7.44, 52.08 (t, ^1J(^{14}N,^{13}C) = 3.0 Hz); $[WO(mnt)_2]^{2-}$, δ 119.54, 141.04. No signals for acetaldehyde or its hydrate were detected.

Based on the known amount of NEt_4^+ inserted with catalyst **1** (2 × 135 μmol), the components

present in the reaction mixture can be quantified by ^{1}H NMR integration as ([W] = 135 µmol or 1 equiv): ethyne (2.0 mmol, 15 equiv), H_2O (3970 equiv. 9.65 mL), MeCN (1156 equiv, 8.16 mL). Compared to the initial amount of solvents (10 mL each), the losses are readily explained by evaporation into the gas flow during the experiment.

b) Static "incubation" experiment

The reaction flask (250 mL Schlenk) was charged with complex **1** (100 mg, 135 µmol), acetonitrile (10 mL) and water (10 mL); the solvents were degassed prior to inserting into the reaction system. Acetylene was bubbled for 30 min through the cooled, dark purple catalyst solution. The acetylene flow was stopped, the flask closed, removed from the ice bath and placed in an oil-bath at 35 °C for 20 h. A sample of 100 µL was removed for direct NMR analysis in (D_6)-DMSO. To the remaining reaction solution, a solution of DNPH (60 mg in 15 mL 2 M HCl aq) was added and the mixture heated for 30 min to 50 °C. After cooling to rt and neutralization with aq 10% Na_2CO_3, a small amount of dark precipitate formed. The mixture was filtered and the solid washed with water. An aliquot of the filtrate was evaporated to dryness. Both the solid and the residue were dissolved in (D_6)-DMSO or $CDCl_3$, respectively, and analyzed by NMR.

NMR analysis of the reaction solution: ^{1}H NMR (500 MHz, (D_6)-DMSO): ethyne, δ 2.68 (s, 2 H); MeCN, δ 2.01 (s); H_2O, δ 3.74 (s); NEt_4^+, δ 1.14 (tt, ^{3}J(H,H) = 7.3 Hz, $^{3}J(^{14}N,^{1}H)$ = 1.9 Hz, 24 H, CH_3), 3.15 (d, ^{3}J(H,H) = 7.3 Hz, 16 H, CH_2). ^{13}C NMR (76 MHz, (D_6)-DMSO): ethyne, δ 75.08 (s); MeCN, δ 1.50, 118.57; NEt_4^+, δ 7.38, 52.00 (t, $^{1}J(^{14}N,^{13}C)$ = 3.1 Hz). No signals for acetaldehyde or its hydrate were detected. The ratio of ethyne (**2**) to tungsten complex **1** in the sample was 11:1.

2.4.2 Experiments using acetylene gas from a commercial pressure gas bottle

a) Blank experiment without catalyst: Acetylene gas from a pressure bottle was slowly bubbled through a mixture of water (10 mL) and acetonitrile (5 mL) in a 25 mL Schlenk vessel. The exhaust gas was bubbled through an acidic (2 M HCl aq) solution of DNPH. After 3 h, the precipitate, which had formed in the DNPH-solution, was isolated by filtration and washed with water to give small amounts of a dark yellow solid identified as acetone dinitrophenylhydrazone (**10**):

^{1}H NMR (400 MHz, $CDCl_3$): δ 2.09 (s, 3 H, Me), 2.18 (s, 3 H, Me), 7.97 (d, ^{3}J(H,H) = 9.6 Hz, 1 H), 8.30 (dd, $^{3,4}J$(H,H) = 9.6, 2.6 Hz, 1 H), 9.13 (d, ^{4}J(H,H) = 2.6 Hz, 1 H), 11.03 (br s, 1 NH).

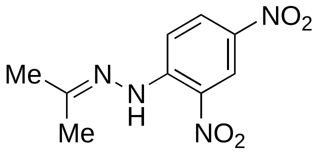

b) Experiment with tungsten catalyst 1

Acetylene gas from a pressure bottle was slowly bubbled through a mixture of water (10 mL) and acetonitrile (5 mL) in a 25 mL Schlenk vessel. Catalyst **1** (23 mg, 31 μmol) was added to give a purple solution. Acetylene was bubbled through the solution for 1 h at 40 °C. Then, the vessel was closed and the reaction kept overnight (14 h) at ambient temperature (20–25 °C). A solution of 2,4-dinitrophenylhydrazine (60 mg, 303 μmol) in 2.4 M HCl aq (15 mL) was added to the (still purple, but darker) mixture. After 30 min, the suspension was filtered, the brown-yellow solid washed with water and dried in air. The material was dissolved in $CDCl_3$ and filtered (from little undissolved dark material) through a cotton plug into an NMR tube. The ^{1}H NMR spectrum showed intense signals for acetone dinitrophenylhydrazone (**10**) and minor peaks for NEt_4^+, but no signals for acetaldehyde derived hydrazone.

2.4.3 Acetylene hydration with a CpRuCl(PPh$_3$)$_2$–ISIPHOS catalyst

The catalyst solution was prepared by placing CpRuCl(PPh$_3$)$_2$ (21.8 mg, 30.0 μmol) and ISIPHOS (14.0 mg, 30.1 μmol) into a 25 mL Schlenk tube under argon, adding degassed triglyme (6.0 mL) and water (1.5 mL) and heating the mixture to 60 °C for 30 min. After cooling to r.t., the catalyst solution was inserted into the usual, argon-flushed reaction setup into the heating block kept at 35 °C. Acetylene was developed and bubbled through the catalyst solution for 4 hours at 35 °C. The exhaust gas was bubbled through a DNPH solution (65 mg DNPH, 15 mL 2 M HCl aq), which became turbid after 2 h and precipitated a considerable amount of solid after 4 h. A sample of the catalyst solution (200 μL) was dissolved in (D$_6$)-DMSO for NMR analysis. The DNPH-suspension was filtered, the solid washed with water and dried in an oven at 60 °C and analyzed by NMR.

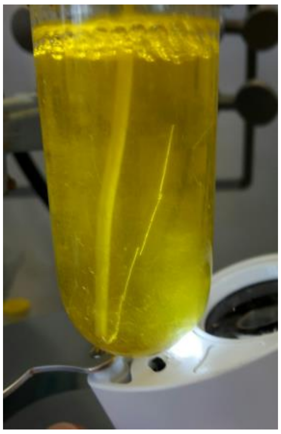

DNPH-solution after 2 h.

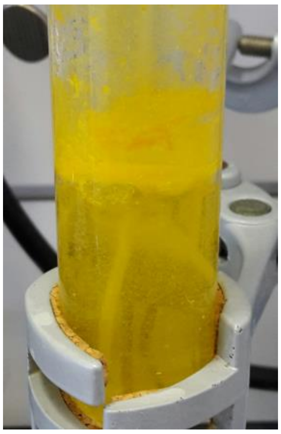

DNPH-solution after 4 h.

NMR analysis of the reaction solution: ^{1}H NMR (400 MHz, (D$_6$)-DMSO): acetaldehyde, δ 2.11 (d, ^3J(H,H) = 2.9 Hz, 3 H), 9.64 (q, ^3J(H,H) = 2.9 Hz, 1 H); ethyne, δ 2.71 (s, 2 H); triglyme, δ 3.23 (s, 6 H), 3.39–3.43 (m, 4 H), 3.47–3.52 (m, 8 H); H_2O, δ 3.53 (s). ^{13}C NMR (101 MHz, (D$_6$)-DMSO): ethyne, δ 74.87 (s); triglyme, δ 58.28, 69.95, 70.16, 71.63, 118.69; the sample was too dilute to detect acetaldehyde by ^{13}C NMR. The molar ratio of acetylene to acetaldehyde of 250:1 (^{1}H NMR)

implies that the majority of aldehyde formed had already been gassed out from the catalyst into the DNPH-solution by the end of the reaction. The concentration of acetaldehyde hydrate was rather low in the triglyme–H_2O mixture and signals for the hydrate could not be clearly discerned.

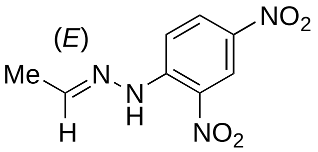

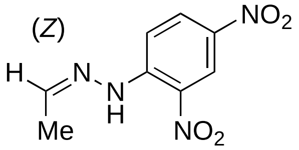

NMR data of acetaldehyde dinitrophenylhydrazone (9): ^{1}H NMR (400 MHz, $CDCl_3$), (*E*)-isomer, 66%: δ 2.14 (d, ^{3}J(H,H) = 5.4 Hz, 3 H), 7.57 (q, ^{3}J(H,H) = 5.4 Hz, 1 H), 7.94 (d, ^{3}J(H,H) = 9.6 Hz, 1 H), 8.30 (ddd, *J* = 9.6, 2.5, 0.6 Hz, 1 H), 9.12 (d, ^{4}J(H,H) = 2.6 Hz, 1 H), 11.04 (br s, 1 H); (*Z*)-isomer, 34%: δ 2.08 (d, ^{3}J(H,H) = 5.6 Hz, 3 H), 7.11 (q, ^{3}J(H,H) = 5.6 Hz, 1 H), 7.97 (d, ^{3}J(H,H) = 9.6 Hz, 1 H), 8.34 (ddd, *J* = 9.6, 2.5, 0.6 Hz, 1 H), 9.14 (d, ^{4}J(H,H) = 2.6 Hz, 1 H), 11.17 (br. s, 1 H). ^{1}H NMR (500 MHz, (D_6)-DMSO), (*E*)-isomer, 75%: δ 2.02 (d, ^{3}J(H,H) = 5.3 Hz, 3 H), 7.85 (d, ^{3}J(H,H) = 9.5 Hz, 1 H), 8.01 (q, ^{3}J(H,H) = 5.3 Hz, 1 H), 8.32 (dd, $^{3,4}J$(H,H) = 9.6, 2.1 Hz, 1 H), 8.83 (d, ^{4}J(H,H) = 2.2 Hz, 1 H), 11.34 (br. s, 1 H); (*Z*)-isomer, 25%: δ 2.04 (d, ^{3}J(H,H) = 5.6 Hz, 3 H), 7.24 (q, ^{3}J(H,H) = 5.4 Hz, 1 H), 7.87 (d, ^{3}J(H,H) $\approx$ 9 Hz, 1 H), 8.39 (dd, $^{3,4}J$(H,H) = 9.6, 2.0 Hz, 1 H), 8.87 (d, ^{4}J(H,H) = 2.1 Hz, 1 H), 10.90 (br s, 1 H). ^{13}C NMR (101 MHz, (D_6)-DMSO), (*E*)-isomer: δ 18.61, 116.24, 123.08, 128.57, 129.78, 136.42, 144.70, 151.46. (*Z*)-isomer: δ 13.47, 116.06, 122.94, 129.55, 130.20, 137.30, 144.78, 148.38.

NMR data of acetaldehyde (3) in D_2O: ^{1}H NMR (250 MHz, D_2O), carbonyl form: δ 2.26 (d, ^{3}J(H,H) = 3.0 Hz, 3 H, CH_3), 9.70 (q, ^{3}J(H,H) = 3.0 Hz, 1 H, MeC*H*O); hydrate: δ 1.35 (d, ^{3}J(H,H) = 5.2 Hz, 3 H, CH_3), 5.26 (q, ^{3}J(H,H) = 5.2 Hz, 1 H, C*H*$(OH)_2$). ^{13}C NMR (63 MHz, D_2O), carbonyl form: δ 29.84 (CH_3), 206.49 (C); hydrate: δ 22.81 (CH_3), 87.85 (CH).

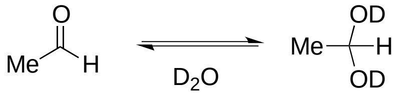

3 NMR spectra

3.1.1 $(NEt_4)_2[WO(mnt)_2]$ (1)

^{1}H NMR (360 MHz, (D_6)-DMSO)

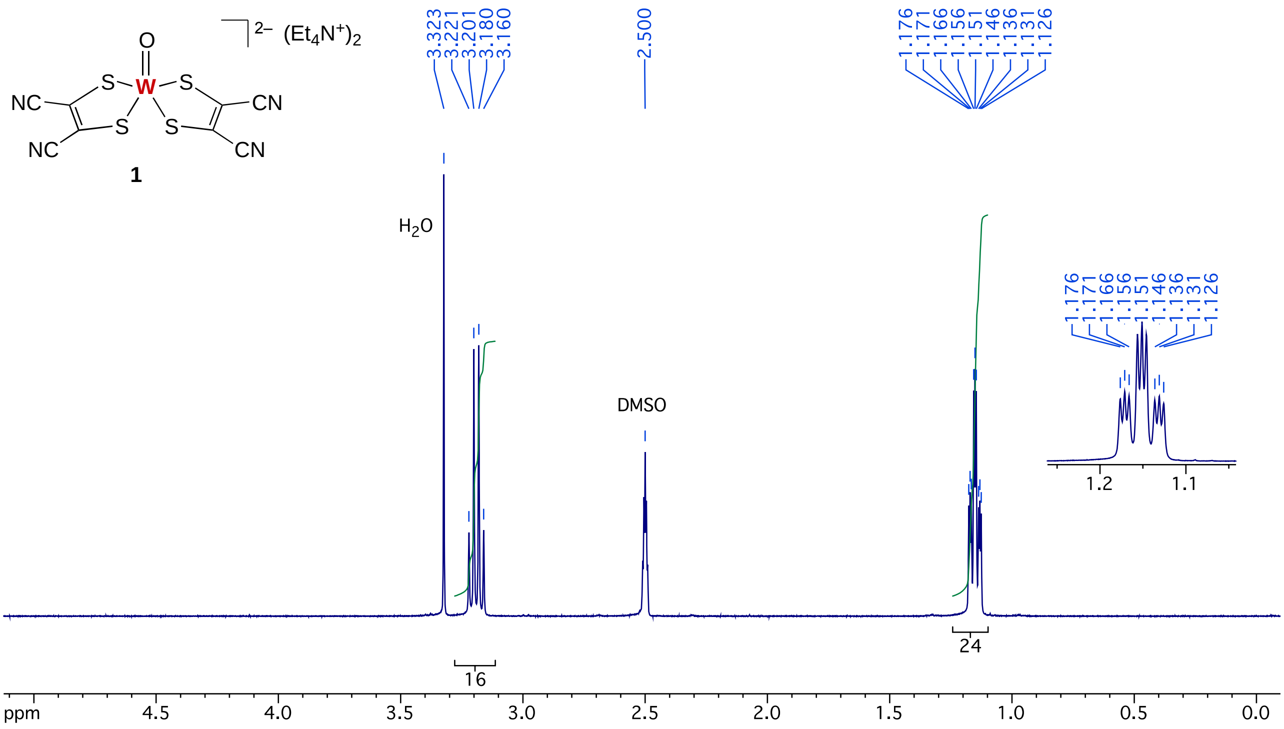

^{13}C NMR (91 MHz, (D_6)-DMSO)

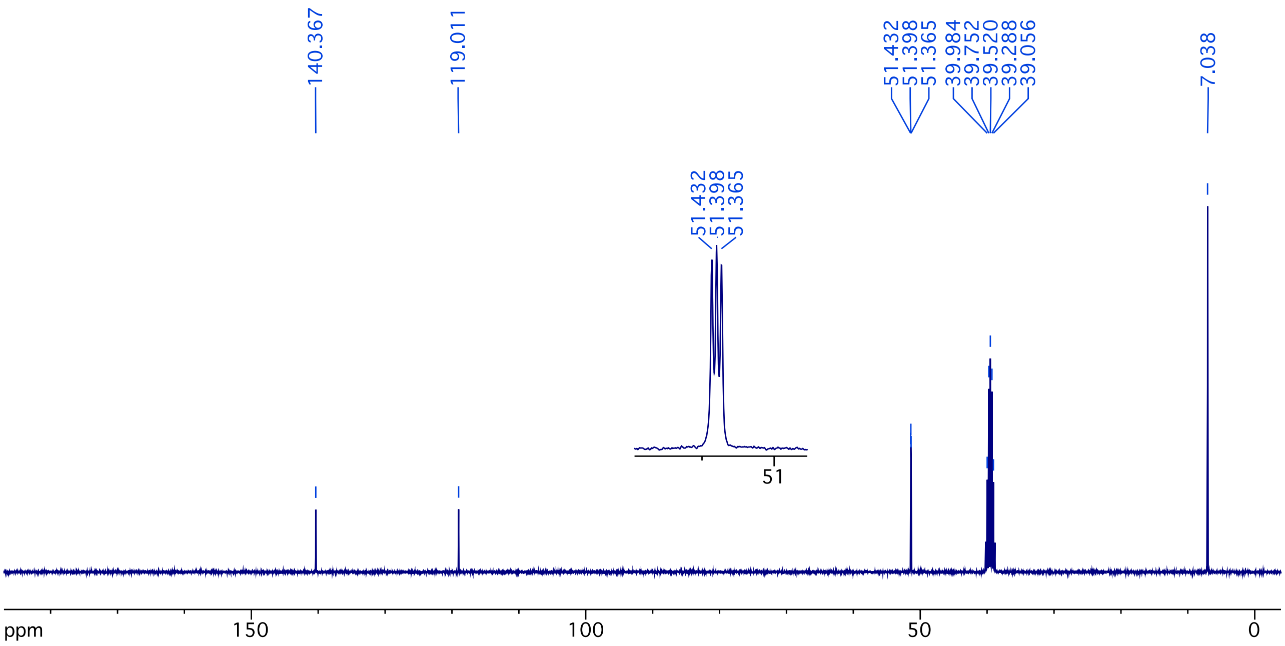

3.1.2 Acetylene (2) hydration experiments

a) Reaction (catalyst) solution of the dynamic experiment with 1 (acetylene bubbling for 4 h)

^{1}H NMR (400 MHz, (D_6)-DMSO)

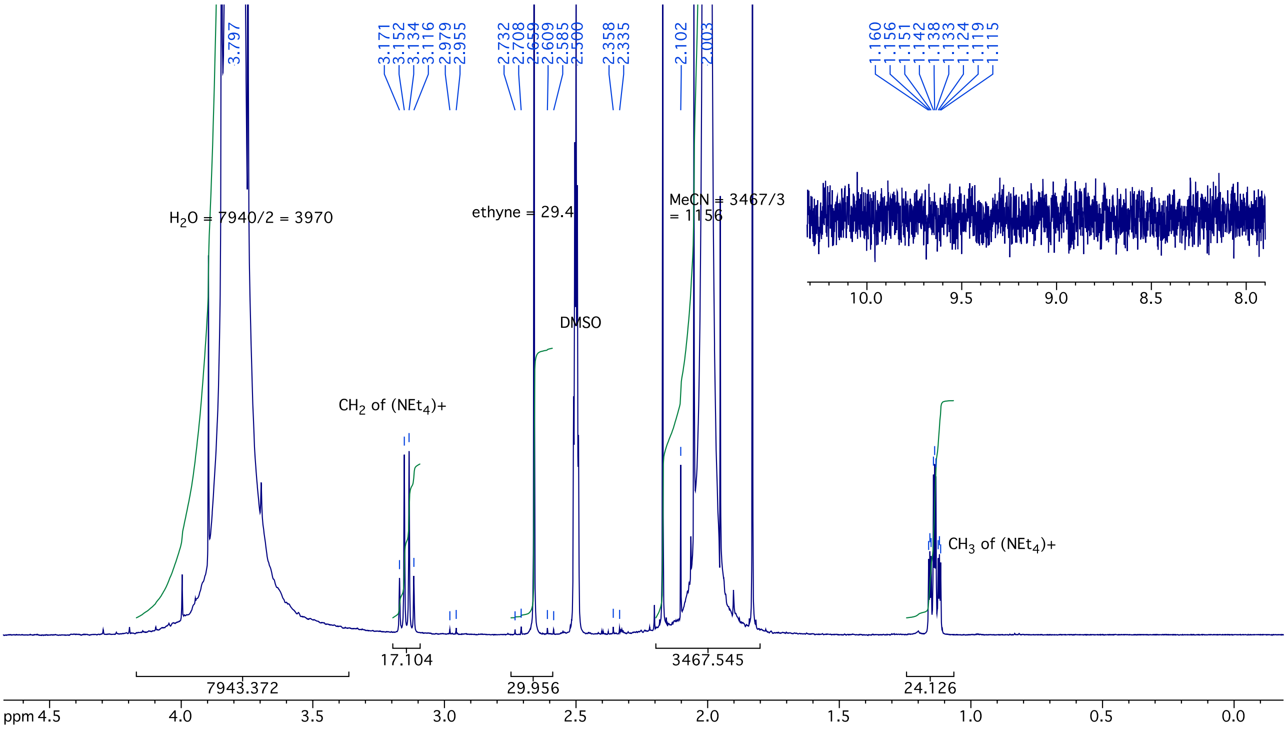

^{13}C NMR (101 MHz, (D_6)-DMSO)

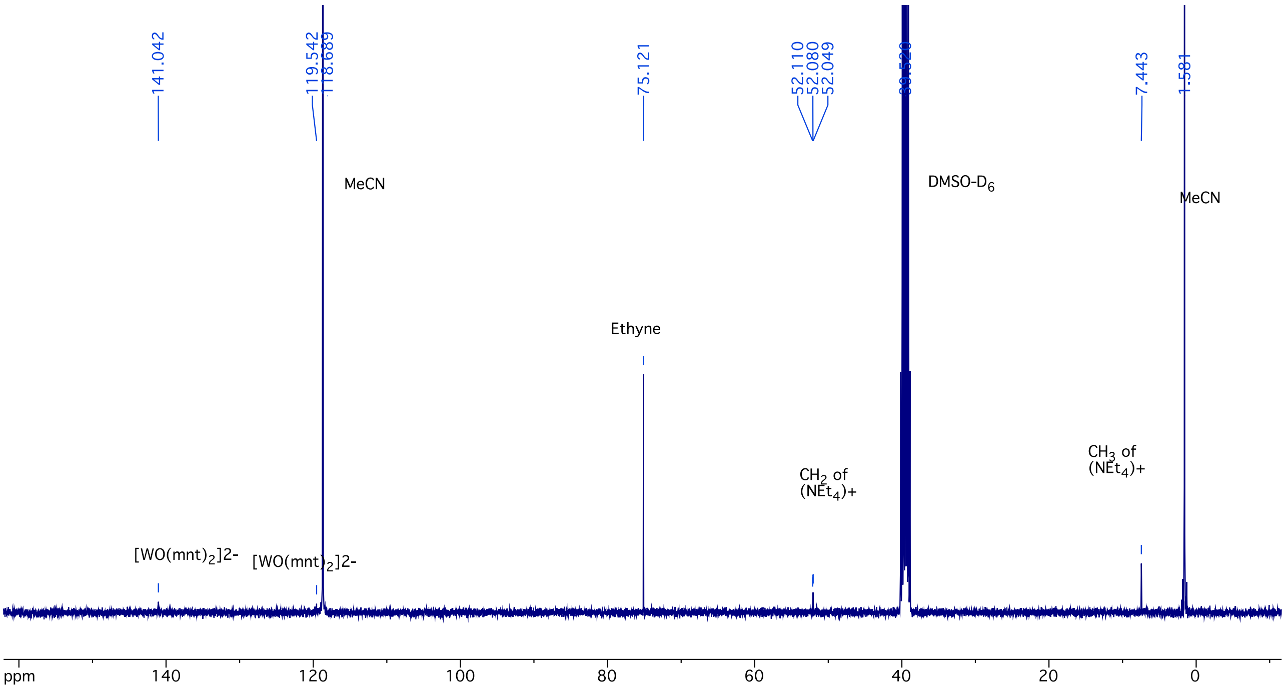

b) Reaction (catalyst) solution of the static experiment with 1 (incubation for 20 h)

^{1}H NMR (400 MHz, (D_6)-DMSO)

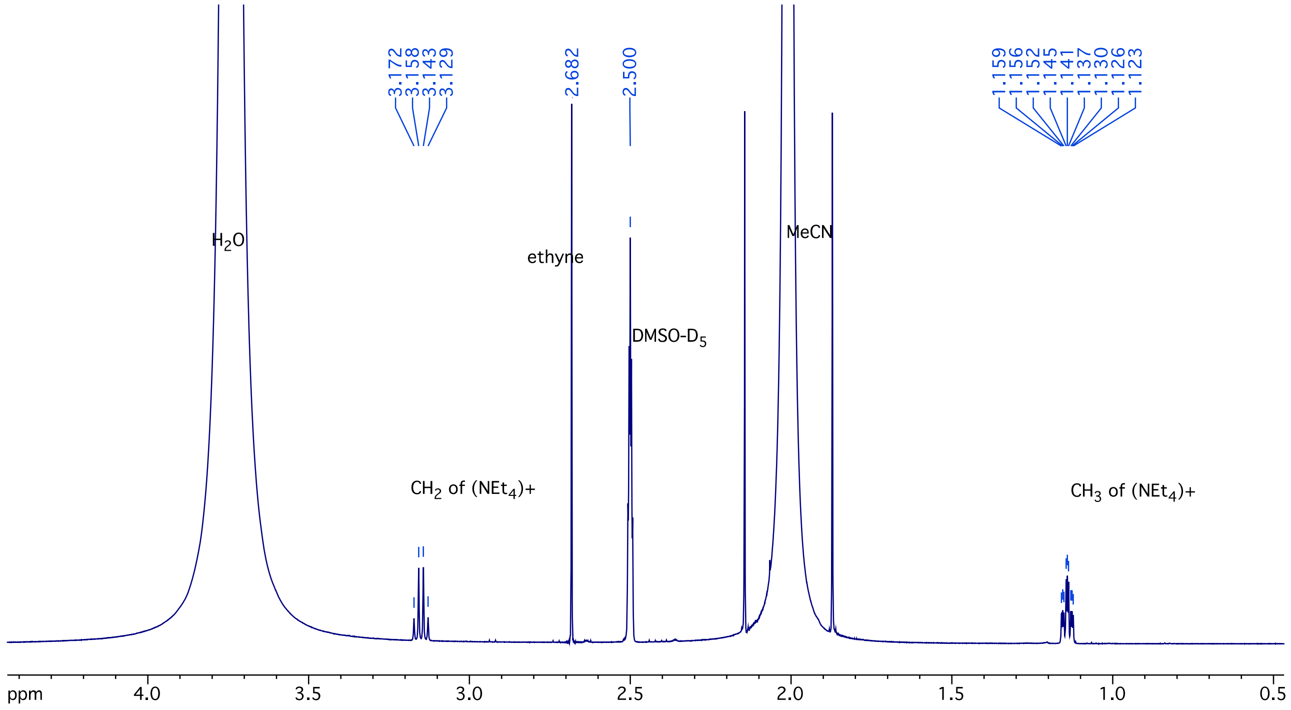

c) Reaction (catalyst) solution of ruthenium-catalyzed acetylene hydration

^{1}H NMR (400 MHz, (D_6)-DMSO); excerpt, solvent and catalyst residual signals cut off

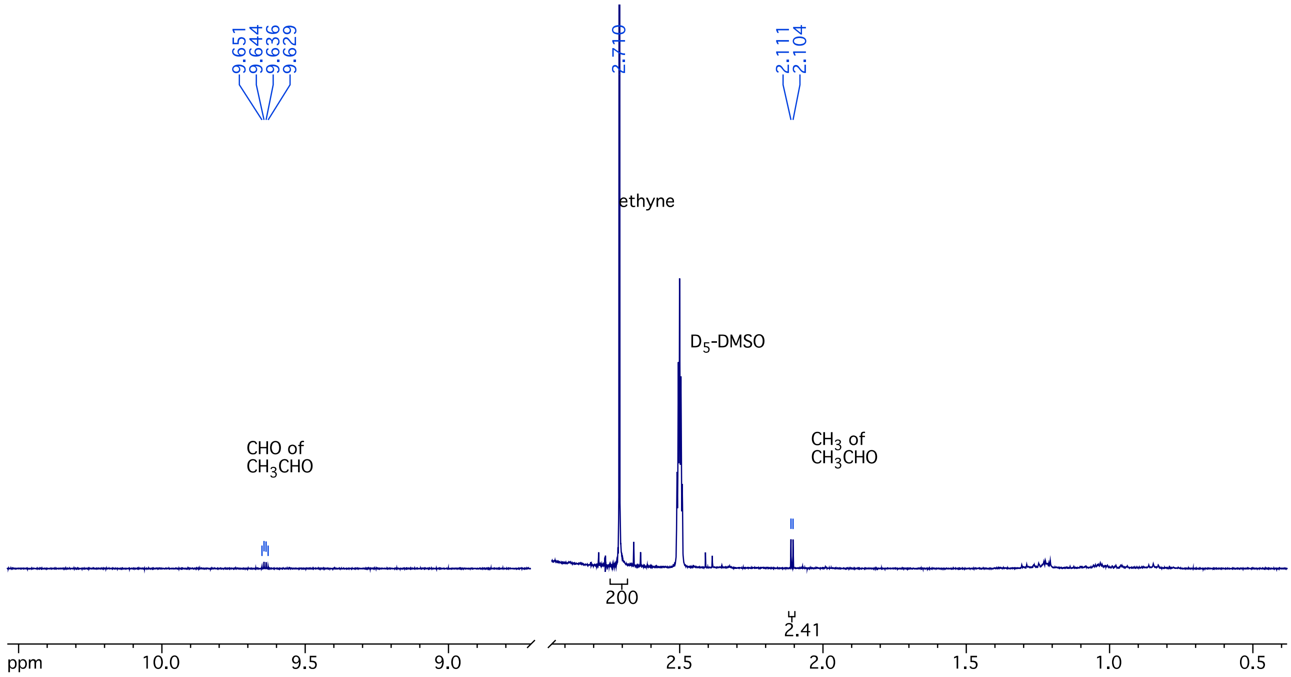

3.1.3 Acetaldehyde (3) reference spectrum in D_2O

^{1}H NMR (250 MHz, D_2O)

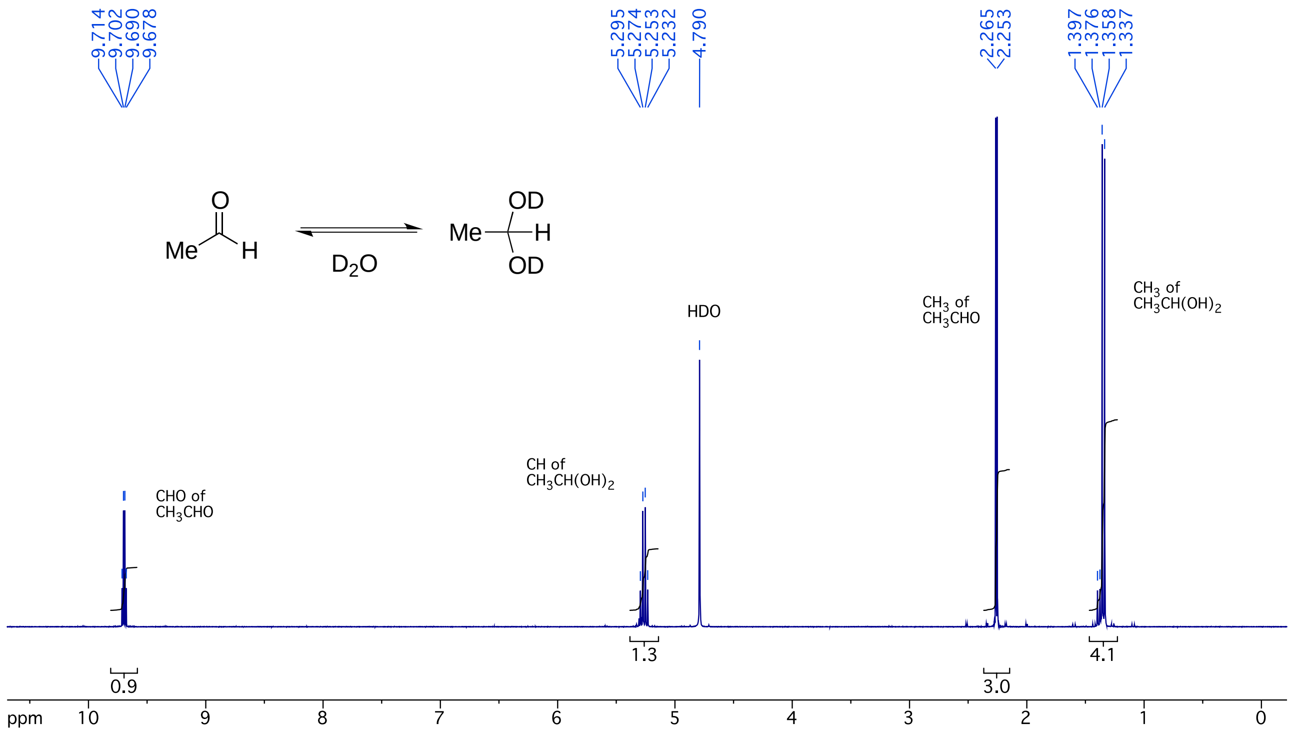

^{13}C NMR (63 MHz, D_2O)

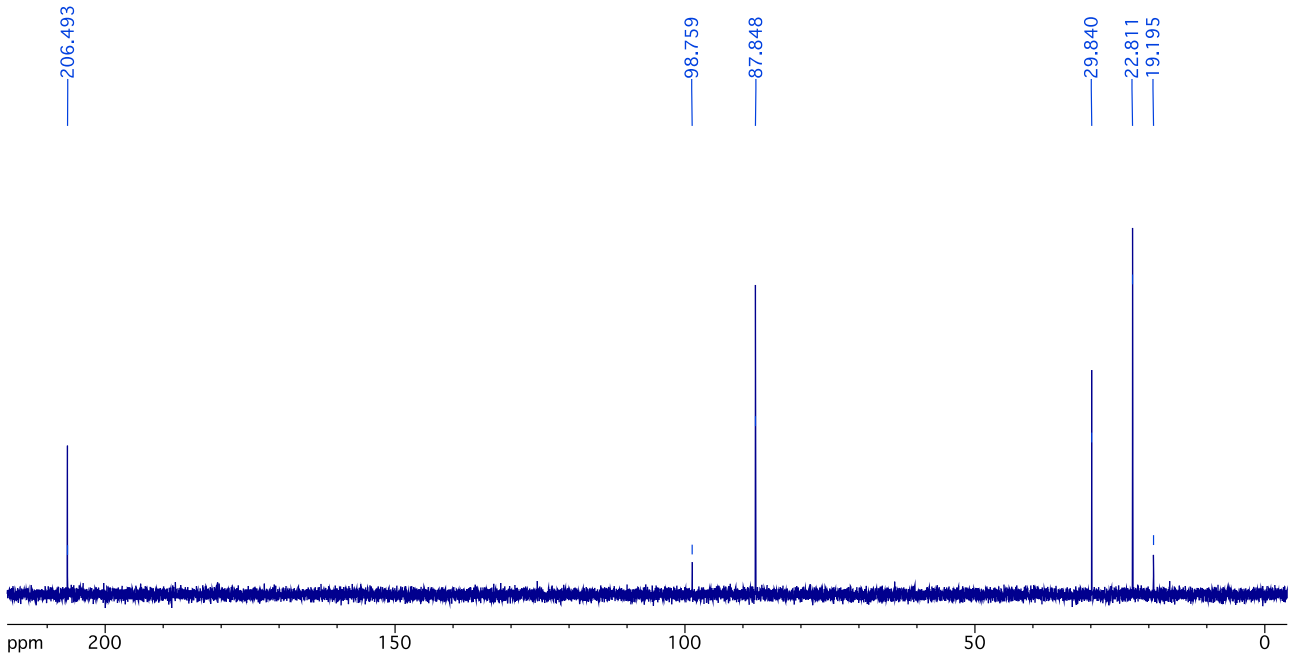

3.1.4 10-Undecyn-1-ol (4)

^{1}H NMR (400 MHz, $CDCl_3$) of distilled material with internal standard

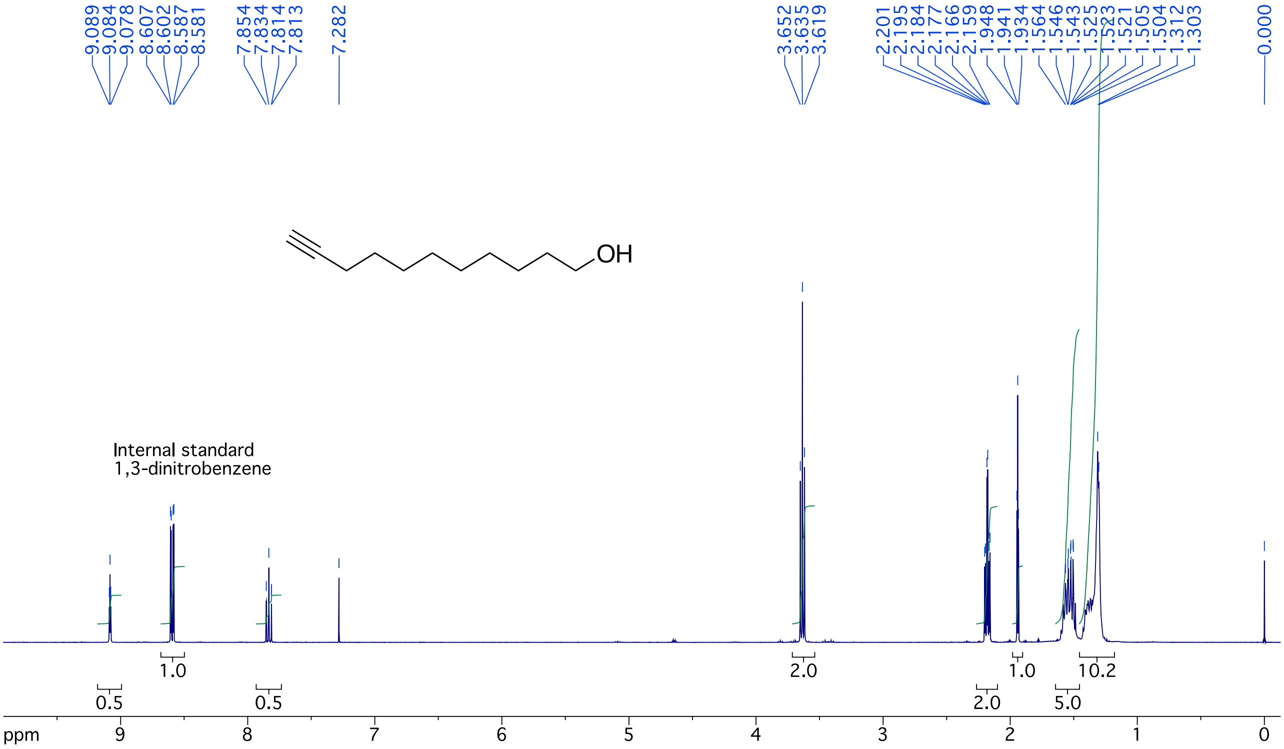

3.1.5 Microwave catalytic hydration screening with AuCl(PPh_3) - crude reaction mixture

^{1}H NMR (500 MHz, $CDCl_3$)

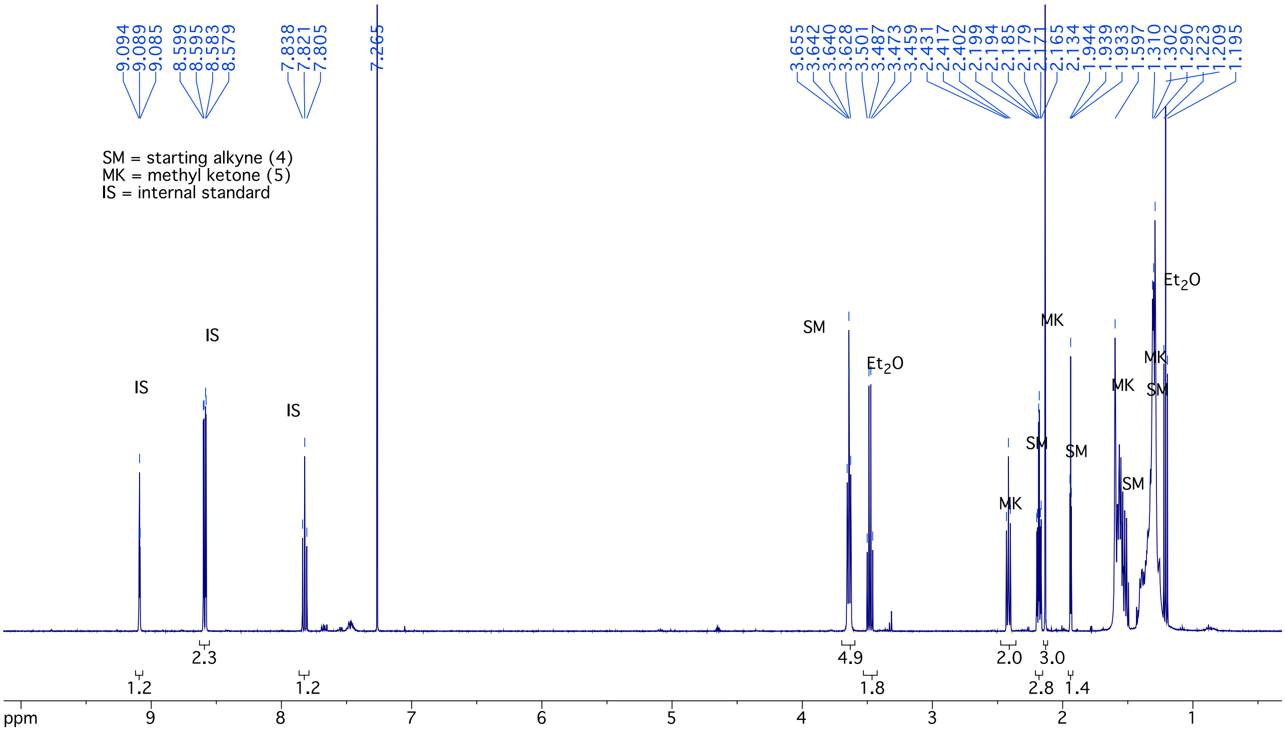

3.1.6 Microwave catalytic hydration screening with CpRuCl(PPh_3)$_2$–ISIPHOS - crude

^{1}H NMR (500 MHz, $CDCl_3$)

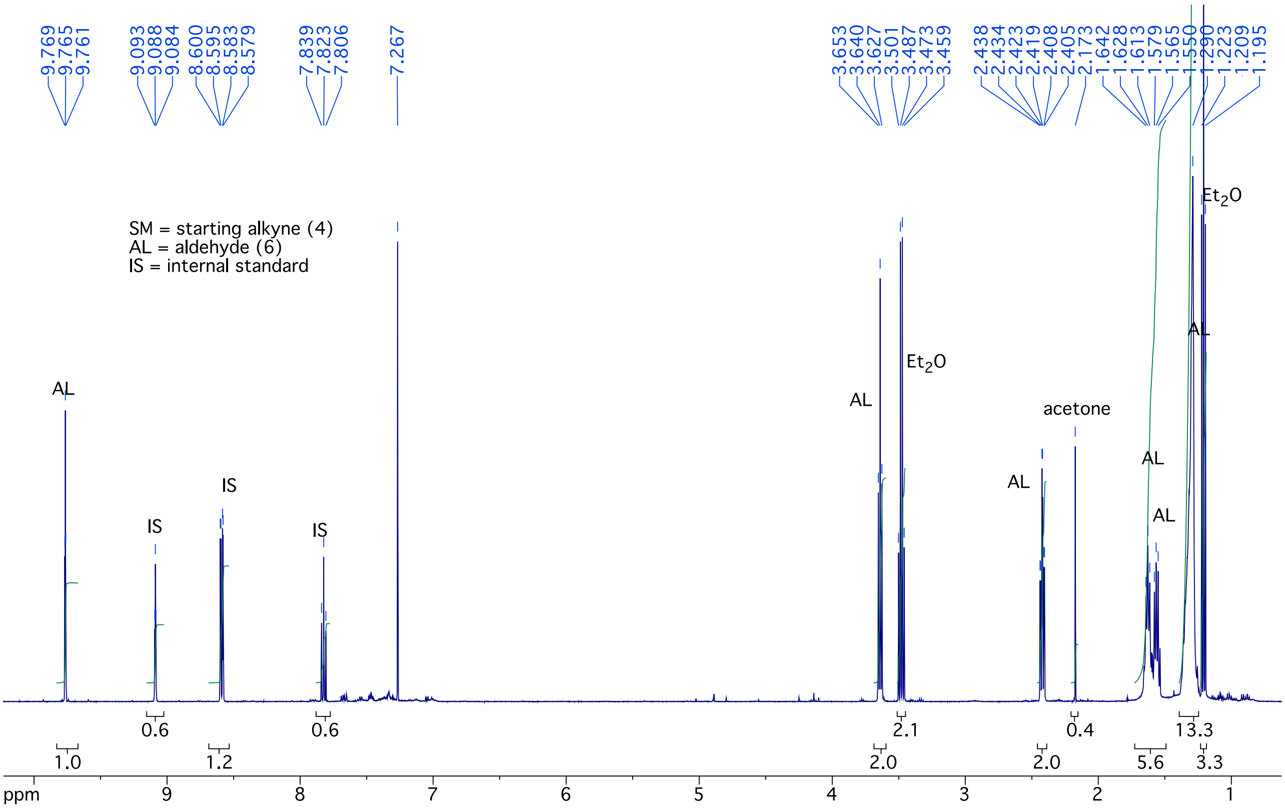

3.1.7 Acetaldehyde 2,4-dinitrophenylhydrazone (9)

^{1}H NMR (400 MHz, $CDCl_3$)

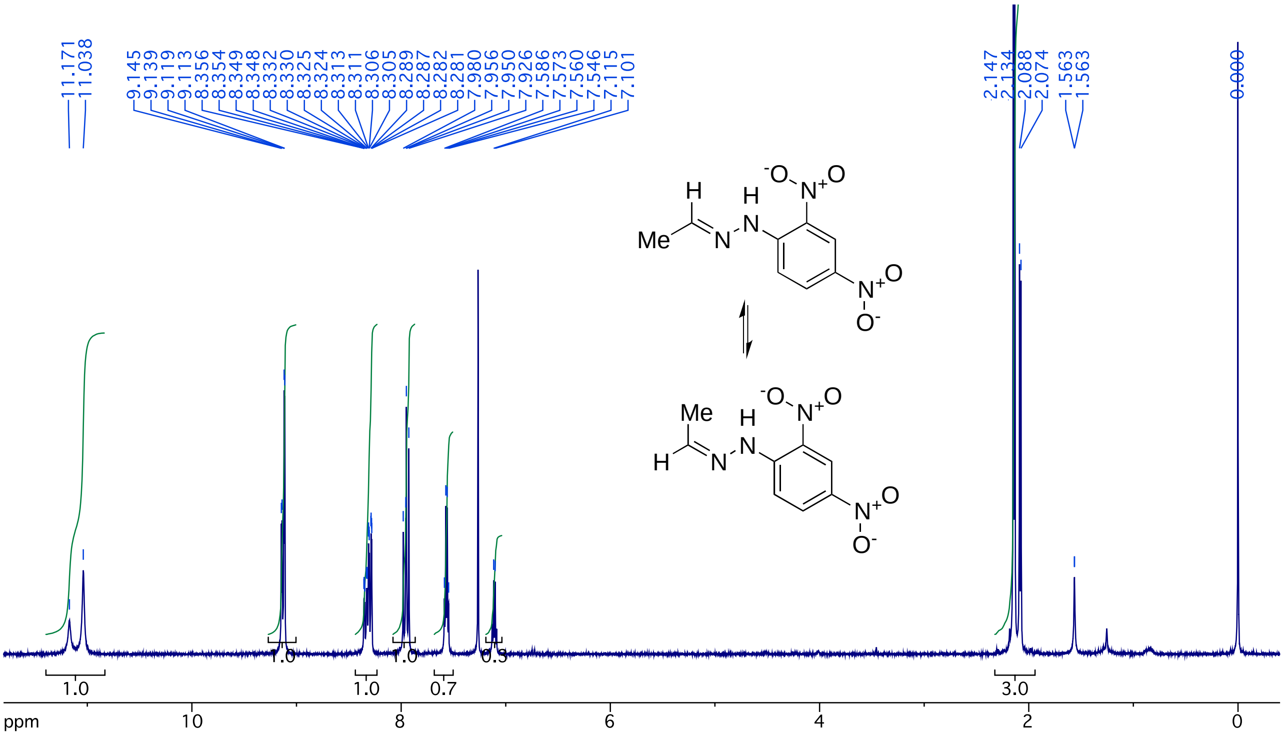

^{1}H NMR (500 MHz, (D_6)-DMSO)

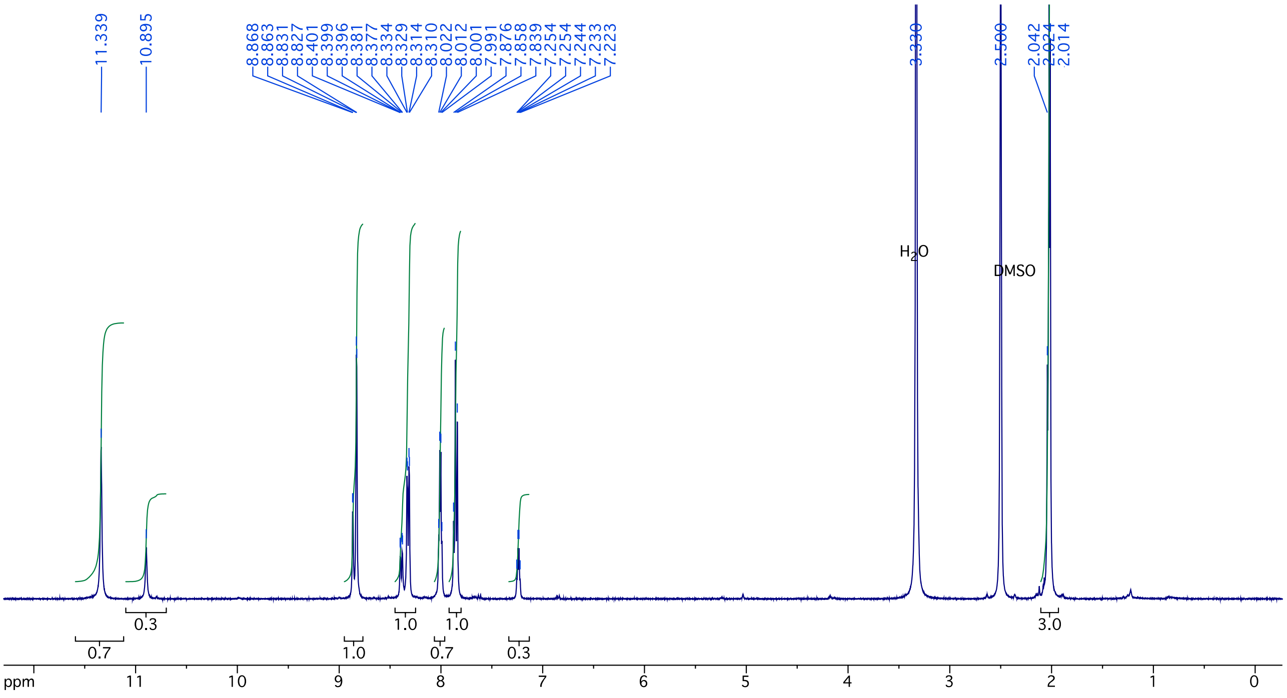

^{13}C NMR (101 MHz, (D_6)-DMSO)

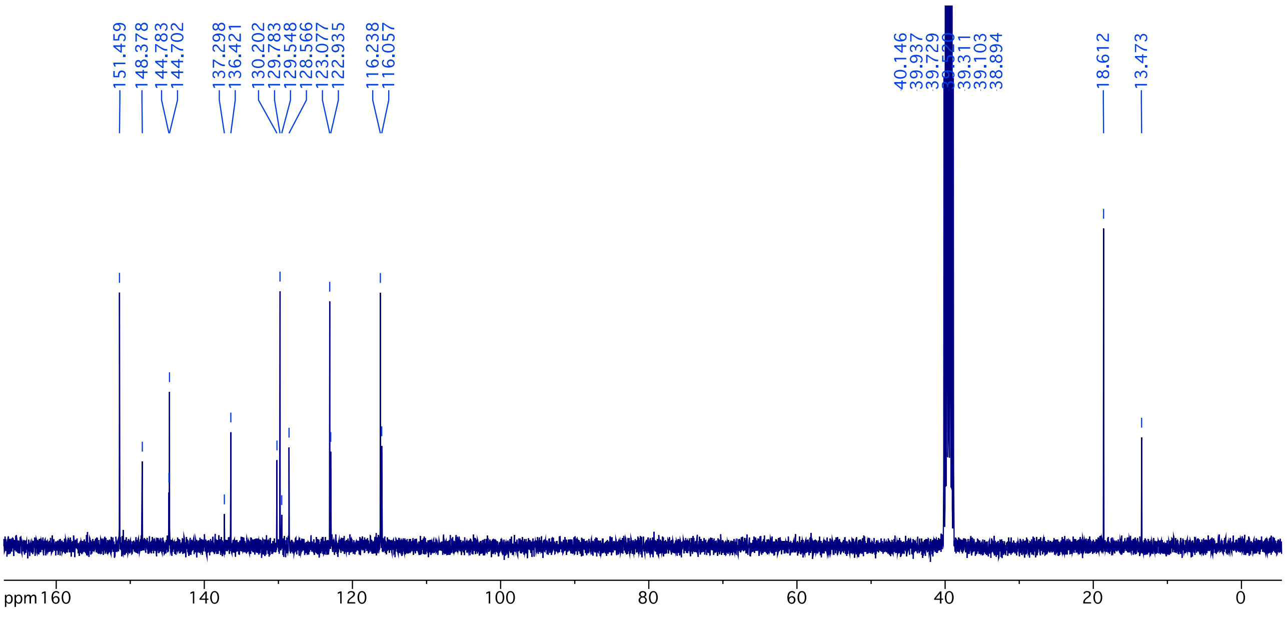

3.1.8 Acetone 2,4-dinitrophenylhydrazone (10)

^{1}H NMR (400 MHz, $CDCl_3$)

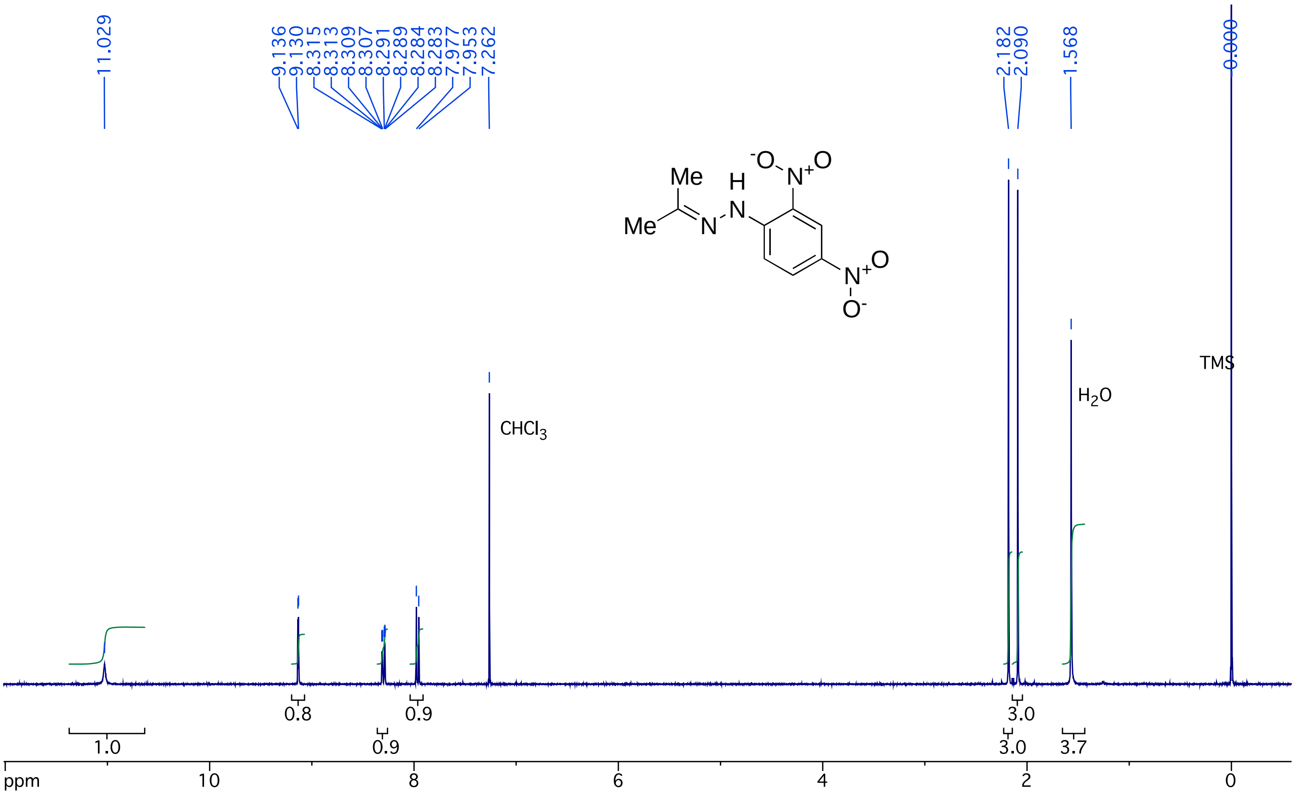

Supplement: File 1 — Experimental procedures and NMR spectra. [file Beilstein_J_Org_Chem-13-2332-s001.pdf]
